# Sequence Analysis of the Genome of Carnation (*Dianthus caryophyllus* L.)

**DOI:** 10.1093/dnares/dst053

**Published:** 2013-12-17

**Authors:** Masafumi Yagi, Shunichi Kosugi, Hideki Hirakawa, Akemi Ohmiya, Koji Tanase, Taro Harada, Kyutaro Kishimoto, Masayoshi Nakayama, Kazuo Ichimura, Takashi Onozaki, Hiroyasu Yamaguchi, Nobuhiro Sasaki, Taira Miyahara, Yuzo Nishizaki, Yoshihiro Ozeki, Noriko Nakamura, Takamasa Suzuki, Yoshikazu Tanaka, Shusei Sato, Kenta Shirasawa, Sachiko Isobe, Yoshinori Miyamura, Akiko Watanabe, Shinobu Nakayama, Yoshie Kishida, Mitsuyo Kohara, Satoshi Tabata

**Affiliations:** 1 NARO Institute of Floricultural Science (NIFS), 2-1 Fujimoto, Tsukuba, Ibaraki 305-8519, Japan; 2 Kazusa DNA Research Institute, 2-6-7 Kazusa-kamatari, Kisarazu, Chiba 292-0818, Japan; 3 Tokyo University of Agriculture and Technology, 2-24-16 Naka-cho, Koganei, Tokyo 184-8588, Japan; 4 Research Institute, Suntory Global Innovation Center, 1-1-1 Wakayamadai, Shimamoto, Mishima, Osaka 618-8503, Japan; 5 JST, ERATO, Higashiyama Live-Holonics Project, Nagoya University, Furo-cho, Chikusa-ku, Nagoya, Aichi 464-8602, Japan; 6 Division of Biological Science, Graduate School of Science, Nagoya University, Furo-cho, Chikusa-ku, Nagoya, Aichi 464-8602, Japan

**Keywords:** *Dianthus caryophyllus* L., carnation, genome sequencing, gene prediction

## Abstract

The whole-genome sequence of carnation (*Dianthus caryophyllus* L.) cv. ‘Francesco’ was determined using a combination of different new-generation multiplex sequencing platforms. The total length of the non-redundant sequences was 568 887 315 bp, consisting of 45 088 scaffolds, which covered 91% of the 622 Mb carnation genome estimated by k-mer analysis. The N50 values of contigs and scaffolds were 16 644 bp and 60 737 bp, respectively, and the longest scaffold was 1 287 144 bp. The average GC content of the contig sequences was 36%. A total of 1050, 13, 92 and 143 genes for tRNAs, rRNAs, snoRNA and miRNA, respectively, were identified in the assembled genomic sequences. For protein-encoding genes, 43 266 complete and partial gene structures excluding those in transposable elements were deduced. Gene coverage was ∼98%, as deduced from the coverage of the core eukaryotic genes. Intensive characterization of the assigned carnation genes and comparison with those of other plant species revealed characteristic features of the carnation genome. The results of this study will serve as a valuable resource for fundamental and applied research of carnation, especially for breeding new carnation varieties. Further information on the genomic sequences is available at http://carnation.kazusa.or.jp.

## 1. Introduction

Carnation (*Dianthus caryophyllus* L.) is one of the major floricultural crops in Japan and worldwide. It is a member of the family Caryophyllaceae and belongs to the genus *Dianthus*. More than 300 *Dianthus* species have been recorded.^1^ Many *Dianthus* species are distributed throughout Europe and Asia, and the distribution of this genus extends to arctic North America and to mountainous sites in Africa.^2^ Several species, including *D. caryophyllus*, *D. barbatus*, *D. chinensis*, *D. plumarius*, *D. superbus* and their hybrids are widely used as horticultural cultivars.^3^ Many new carnations have been bred for attractive characteristics such as flower colour, flower size, fragrance and flower longevity.

The pigments in carnation flowers are mainly anthocyanin and chalcone derivatives, and most of the genes involved in pigment biosynthesis in carnation have been identified.^4^ Due to the absence of flavonoid 3′,5′-hydroxylase (F3′,5′H; a key enzyme in the synthesis of delphinidin) in carnation, blue or violet flowers have never occurred in carnations. The introduction of a petunia or pansy *F3′,5′H* gene into carnation has led to the creation of blue or violet transgenic carnations, which are commercially available.^5^ The plant pigments of species belonging to the families of Caryophyllales (except for Caryophyllaceae and Molluginaceae) are betalains, which have never been detected with anthocyanins in the same species.^6^ The carnation, exceptionally bearing anthocyanins in Caryophyllales, is one of the attractive materials to study evolution of genetic systems for pigment synthesis.

The vase life of cut flowers, or flower longevity, is one of the most important characteristics to carnation.^7^ Carnation flowers are highly sensitive to ethylene, which induces autocatalytic ethylene production and wilting in carnation petals.^8^ Conventional cross-breeding techniques have succeeded in improving the vase life of the carnation flower,^9^ which is a polygenic trait that is controlled by several genes involved in ethylene production and ethylene sensitivity.^9,10^

To clarify the genetic and physiological mechanisms of agriculturally important traits, and to apply this information to actual breeding, a number of genetic and molecular tools have been developed. Genetic linkage maps of the carnation genome have been constructed and used to identify quantitative trait loci (QTL) responsible for resistance to carnation bacterial wilt.^11,12^ With the aid of next-generation sequencing (NGS) technology, large-scale transcriptome analysis (RNA-seq) has been conducted, revealing 300 740 unigenes consisting of 37 844 contigs and 262 896 singletons.^13^ Recently, we constructed a reference genetic linkage map for carnation using simple sequence repeat (SSR) markers derived from this RNA-seq analysis.^14^

Most carnation cultivars are diploid, with a chromosome number of 2*n* = 2*x* = 30.^15^ The reported nuclear DNA content of carnation is 1.23–1.48 pg/2C, which indicates that the carnation has a comparatively small nuclear genome approximately four times the size of the *Arabidopsis thaliana* nuclear genome.^16^ The estimated genome size of carnation (670 Mb)^16^ is small compared with those of other ornamental flowers, such as *Rosa hybrida* (1.1 Gb), *Antirrhinum majus* (1.5 Gb), *Petunia hybrida* (1.6 Gb), *Chrysanthemum morifolium* (9.4 Gb) and *Tulipa gesneriana* (26 Gb), according to the Plant C-values database (http://data.kew.org/cvalues/). To understand the genetic systems of carnation and to accelerate the process of molecular breeding, we performed structural analysis of the whole genome of carnation for the first time in ornamentals. The information and material resources for the carnation genome generated in this study should enhance both fundamental and applied studies of carnations and related plants.

## 2. Materials and methods

### 2.1. Plant materials

The carnation cultivars, ‘Francesco’ (for genome sequencing) and ‘Karen Rouge’ (for BAC construction) were grown under natural daylight conditions in a greenhouse in NIFS. ‘Francesco’, a red Mediterranean standard-type cultivar, is the leading cultivar in Japan,^17^ and ‘Karen Rouge’ is a cultivar with bacterial wilt resistance derived from *D. capitatus* ssp. *andrzejowskianus*.^18^

### 2.2. Construction of BAC libraries and BAC DNA sequencing

BAC libraries were constructed from nuclear DNA prepared from young leaves of ‘Karen Rouge’. Nuclear DNA was partially digested with *Hin*dIII and size-selected, and 100–180 kb DNA was ligated to the BAC vector pIndigoBAC5 (Epicentre Biotechnologies, WI, USA) and introduced into *Escherichia coli* ElectroMAX DH10B cells (Life Technologies Co., CA, USA) by electroporation.

For shotgun sequencing of BAC clones, BAC DNAs prepared according to the standard procedure were fragmented by nebulization, barcoded with a GS Titanium Rapid Library MID Adaptors Kit (Roche Diagnostics, IN, USA), and pooled for sequencing using the GS Titanium platform (Roche Diagnostics) according to the manufacturer's instructions.

### 2.3. Shotgun sequencing of the carnation genome

Whole-genome shotgun sequencing of the cultivar ‘Francesco’ was performed using both HiSeq 1000 (Illumina Inc., CA, USA) and GS FLX+ (Roche Diagnostics) sequencers. Genomic DNA extracted from leaves was used for library construction according to standard protocols. The libraries included paired-end (PE) (insert size: 500 bp) and overlapping fragment (OF) (insert size: 180 bp) libraries for the HiSeq 1000 sequencer, and single-end (SE) and PE libraries (insert size: 4 kb) for the GS FLX+ system. In addition, two Illumina mate-pair (MP) libraries with 3 and 5 kb inserts, respectively, were constructed with GS Titanium Library Paired End Adaptors (Roche Diagnostics) as previously described.^19,20^ The sequence data collection is summarized in Supplementary Fig. S1.

### 2.4. Sequence assembly and evaluation of authenticity

A method taken for assembly of the genomic sequences of carnation is summarized in Supplementary Fig. S1. Sequence assembly for the BAC DNAs was performed using Newbler ver. 2.7 (Roche Diagnostics). Authenticity of the assembled genomic sequences described above was examined by aligning the sequences of subcontigs contained in the scaffolds of the genomic assemblies with the contig sequences of the BAC clones using the Bowtie 2 program. References for computer programs and databases are listed in Supplementary Table S1.

### 2.5. cDNA sequences

Carnation cDNA sequences generated by Sanger sequencing (accession numbers: FY382825–FY405424)^12^ and GS FLX+ sequencing (accession numbers: FX296474–FX334317)^13^ were retrieved from NCBI GenBank (http://www.ncbi.nlm.nih.gov/genbank/). In order to generate a non-redundant cDNA data set, redundant cDNA sequences were removed with a CD-HIT tool.

### 2.6. Detection of repetitive sequences

Known repetitive sequences, including transposable elements (TEs), were detected with the RepeatMasker (http://www.repeatmasker.org) and TransposonPSI (http://transposonpsi.sourceforge.net) programs, and novel repeats were detected with RepeatScout and Piler. To exclude protein-coding genes, the novel repeat library was searched against the SWISS-PROT database (http://www.uniprot.org) with BLASTX. The known and novel repeat sets were merged and redundant repeats were removed.

### 2.7. Assignment of RNA-coding genes

Genes for tRNAs were assigned using the tRNAscan-SE program. The rRNA genes were identified based on sequence similarity with those of *A. thaliana*. Genes for small nucleolar RNA (snoRNA) were predicted using snoScan. Micro RNA (miRNA) genes were searched against a miRBase library that contained plant miRNA sequences detected by the MapMi program, which assigns miRNA precursor sequences in genomes by a combination of sequence alignment with Bowtie and prediction of RNA-secondary structures with RNAfold. References for computer programs and databases are listed in Supplementary Table S1.

### 2.8. Assignment of protein-encoding genes

Two programs were used for the assignment of protein-encoding genes in the carnation genome: PASA (http://pasa.sourceforge.net), based on cDNA alignment and Augustus, based on *ab initio* gene prediction incorporating cDNA alignment information. The protein-encoding genes were first predicted using PASA and then, Augustus was trained with a dataset comprising 300 PASA-predicted genes likely containing complete coding regions. The trained Augustus was implemented with a hint file, which was generated by alignment of the cDNA set to the genome with BLAT. Finally, both the PASA- and Augustus-predicted datasets were merged, and only genes with a single exon predicted in both datasets, not in either one of the two datasets, were selected because the prediction accuracy of genes with a single exon is generally low.

To deduce coding regions of the predicted genes, all possible amino acid sequences translated from three reading frames for multiple exon genes and six reading frames for single exon genes were similarity-searched against the Uniprot-Tremble database (http://www.ebi.ac.uk/uniprot/) with BLASTP. Translated amino acid sequences that had a similarity to a protein in the database with *E*-value ≤ 1*E*–5, identity ≥ 30% and minimal length ≥ 50 amino acids were selected and defined as the coding regions. When all translated sequences of a gene exhibited no similar proteins, the longest coding region was selected. To confirm the coverage of the assembly and the accuracy of the annotated genes, core eukaryotic genes were mapped using CEGMA. References for computer programs and databases are listed in Supplementary Table S1. The predicted genes related to TEs were excluded for further analyses.

### 2.9. Comparison of metabolic pathways

For comparison of the metabolic pathway, *Beta vulgaris*, *A. thaliana* and *Oryza sativa* were chosen. *B. vulgaris,* which has numerous cultivated root vegetables such as table beet and sugar beet, is a member of the same order Caryophyllales with carnation and has relatively large number of registered genes among this order. *Arabidopsis thaliana* and *O. sativa* are typical models of dicot and monocot, respectively. The nucleotide sequences of gene repertoires of *A. thaliana* and *O. sativa* were retrieved from the genome databases of TAIR10 and IRGSP 1.0, respectively. For *B. vulgaris*, expressed sequence tag (EST) sequences were obtained from dbEST of the NCBI databases and trimmed using the CROSS_MATCH program for vector sequences provided in CROSS_MATCH and NCBI's Univec (http://www.ncbi.nlm.nih.gov/tools/vecscreen/univec/) reads longer than 100 bp were subjected to assembly by PHRED with default parameters. The sequences of genes and unigenes thus obtained, as well as those of the predicted carnation genes, were mapped onto the KEGG reference pathways by BLAST searches against genes in the KEGG database with *E*-value cutoff of 1*E*–10, length coverage ≥25% and identity ≥50%, and the status of mapping was compared among the four plant species. References for computer programs and databases are listed in Supplementary Table S1.

### 2.10. Functional classification of genes

The predicted gene sequences of carnation were searched against NCBI's Clusters of Orthologous Groups of proteins (KOG) by BLAST searches with *E*-value cutoff of 1*E*-20. In addition, functional domains located in the translated sequences in genes and unigenes were searched against the InterPro databases using InterProScan, and the detected domains were further classified into plant GO slim categories using the map2slim program.

## 3. Results and discussion

### 3.1. Sequencing the carnation genome

#### 3.1.1. Shotgun sequencing and assembly of the carnation genome

Shotgun sequencing of the genome of the carnation cv. ‘Francesco’ was carried out using a combination of different sequencing libraries for two NGSs, the HiSeq 1000 and the GS FLX+ systems. In the HiSeq 1000 system, a total of 1277.4 million (M), 1526.5, 442.6 and 475.3 M reads corresponding to 127.7, 152.6, 44.3 and 47.5 Gb sequence data were collected from the PE, OF, 3 kb MP and 5 kb MP libraries, respectively (Supplementary Fig. S1). In parallel, 5.9 M single reads (mean length: 663 bases) and 1.3 M PE reads (mean length: 436 bases) corresponding to 3.9 Gb and 589 Mb sequence data, respectively, were obtained using the GS FLX+ system (Supplementary Fig. S1). The genome size of carnation cv. ‘Francesco’, which was estimated by k-mer analysis^21^ based on the HiSeq 1000 sequence data, was 622 Mb, which is 93% of the previous estimate (670 Mb)^16^ this value was adopted for subsequent analyses. Total redundancy of the obtained sequence data (376.6 Gb) was equivalent to ∼604-times the estimated genome size.

The method used for genomic data assembly is described in the Materials and methods and summarized in Supplementary Fig. S1 and Table S1. The total length of the resulting genomic assemblies was 568.9 Mb, equivalent to 91% of the estimated genome size, containing 69 Mb gaps filled by N; the N50 values of the contigs and scaffolds were 16 644 bp and 60 737 bp, respectively (Table 1).

**Table 1.** Statistics of *D. caryophyllus* cv. ‘Francesco’ genome assemblies

	Limited length^a^	Contigs^b^	Scaffolds
Total number	≥100 bp	88 654	45 088
Total length (kb)		50 0218	568 887
N50 (bp)		16 644	60 737
N90 (bp)		2036	6700
Total number	≥1 kb	64 159	30 716
Total length (Mb)		483 703	558 518
N50 (bp)		17 553	62 616
N90 (bp)		2678	8036
Maximum length (kb)		363	1287
GC content (%)		36.3	

^a^Contigs or scaffolds shorter than the indicated length were excluded from the statistics.

^b^Subcontigs in scaffolds, which were split by gap regions with length >4 bp.

The N50 value of the final scaffolds was relatively low, probably due to the heterozygotic nature (∼0.2% heterozygosity estimated in this study; data not shown) of the carnation genome. Because current methods for *de novo* assembly using de Bruijn graphs split assemblies at heterologous polymorphic sites, assemblies of heterozygous diploid genomes tend to be fragmented. However, the N50 value of the contigs contained in the scaffolds was 16.6 kb (Table 1), which allowed reliable characterization and gene annotations. Mapping of 248 core eukaryotic genes by the CEGMA program indicated that 96% of the core genes were completely covered in the genome assemblies. Comparison of the independently determined sequences of the two BAC clones with the assembled genomic sequences showed perfect alignment with correct order and coverage, demonstrating that the coverage and quality of the assembled genomic sequences were high.

#### 3.1.2. Correlation of the genomic sequences with a genetic linkage map

We constructed an SSR-based reference genetic linkage map of the carnation genome comprising 412 SSR loci on a total length of 969.6 cM.^14^ To correlate the genomic sequences obtained in this study to their positions on this linkage map, we searched the assembled genomic sequences for sequences of these markers and their adjacent regions using the BLASTN program. All primer sequences, sequences of the flanking regions and SSR motifs were successfully mapped to the assembled genomic sequences, although there were single base substitutions or small deletions of several bases long. Single corresponding scaffolds could be identified for 378 (91.7%) of the 412 SSR loci (Supplementary Table S2), and the remaining SSR loci were assigned to multiple scaffolds containing identical or highly similar sequences. Consequently, 268 scaffolds could be located on the genetic linkage map. Some of the scaffolds covered multiple marker loci, e.g. scaffold6 on LG 85P_5, with six SSR loci. The longest mapped scaffold was scaffold2 on LG 85P_9, which covered 1.2 Mb regions and three marker loci. The total length of the mapped scaffolds was 51.4 Mb, equivalent to 8.3% of the estimated genome size.

### 3.2. Characteristic features of the carnation genome

#### 3.2.1. Repetitive sequences

The repetitive sequences found in the assembled genomic sequences comprised 112 078 known TEs, 354 221 simple repeats and low complexity sequences, and 331 831 novel repeats defined by *de novo* repeat finding, the sum of which correspond to 33% of the assembled genomic sequences (Table 2). Comparison of the TE contents of the carnation genome with those of other plant species, such as *A. thaliana*, *Brassica rapa*, potato, tomato, cotton, soybean, sweet orange, rice, foxtail millet and sorghum (Supplementary Table S3), showed that the relative content of known TEs was lower than those of unclassified repeats, and simple repeats appeared to be higher in carnation. The lower content of TEs in the assembled genomic sequences in carnation may be attributed to the escape of TE sequences during the process of sequence assembly, since the carnation assemblies in this study were highly fragmented at the positions of potential repetitive sequences.

**Table 2.** Repetitive sequences identified in the carnation genome

Repeat class	Number	Total length (kb)	Genome content (%)^a^	Repeat composition (%)
Class 1 TE				
LTR	86 111	36 558	7.3	22.1
SINE	441	35.7	0.007	0.02
LINE	7362	3447	0.69	2.1
Other	10	1.7	0.0003	0
Class 2 TE				
DNA transposon	16 729	3914	0.78	2.4
Rolling circle	1425	535	0.11	0.33
Other type TE	48	21.1	0.004	0.01
Simple repeat	295 621	16 847	3.4	10.3
Low complexity	58 600	3528	0.71	2.2
Other repeat	411	65	0.004	0.01
Unclassified (novel) repeat	331 831	100 097	20	60.6
Total	794 297	165 027	33	100

LTR, long terminal repeat; SINE, short interspersed nuclear element; LINE, long interspersed nuclear element.

^a^Percentage of total length of repeats in the continuous sequences in the assembled genome.

In carnation, the mechanism underlying variegated flower colour has long been of major horticultural interest.^22^ An excision event of Class II DNA TEs has been identified from the genes for enzymes involved in anthocyanin biosynthesis.^23^ *Tic101*, a member of *hAT* (*Ac*/*Ds*) elements, was identified as an autonomous TE encoding an active transposase protein.^4^ A single homologue of *Tdic101*, designated *Tic104*, whose sequence is 99% (Supplementary Table S4) identical to that of *Tdic101* was detected in the assembled genomic sequences.^4^ *Tic104* has one nucleotide substitution in a terminal-inverted repeat sequence and another in the coding region of the transposase gene to generate a stop codon (Supplementary Fig. S2). The CACTA element (*En*/*Spm*), *dTac1* was first identified in a gene for glutathione-*S*-transferase, here designated *Tac101*, which is not likely to encode notable proteins for transposases.^24^ A similarity search against the ‘Francesco’ genomic sequences detected four types of CACTA elements, designated *Tac201*, *Tac301*, *Tac401* and *Tac501*, in addition to *Tac101* (Supplementary Table S4 and Fig. S3). None of these is likely to encode intact transposases that are active for transposition.

The genome of ‘Francesco’ contains an *acyl-glucose-dependent anthocyanin 5-glucosyltransferase* (*AA5GT*) gene with an insertion by *Ty1-1* (*Ty1dic1*), resulting in synthesis and accumulation of pelargonidin 3-*O*-malylglucoside that lacks its glucose moiety at the 5 positions. *Ty1-1* in the ‘Francesco’ genome has one nucleotide substitution in the coding region of the transposase gene to generate a stop codon different from *Ty1dic1*. Similarity searching of the assembled genomic sequences indicated that there were six genes for *Ty1-1* longer than 1 kb (Supplementary Table S4 and Fig. S4).

#### 3.2.2. Genes coding for RNAs

Thirteen genes for rRNAs and 1050 intact genes for tRNAs were identified in the assembled genomic sequences. Comparison of the number of genes for tRNAs in the genomes of 16 plant species indicated that the carnation genome contains many more genes than other plant species (Supplementary Table S5). A total of 92 and 143 genes for snoRNA and miRNA, respectively, were also assigned.

#### 3.2.3. Prediction of protein-encoding genes

We assigned protein-encoding genes in the carnation genome using two types of computer programs, PASA and Augustus. As a result, 10 519 protein-encoding genes predicted by PASA and 99 123 genes predicted by Augustus were merged, resulting in assignment of 56 137 protein-encoding genes including those in TEs (Supplementary Table S6).

A similarity search against the Uniprot-Tremble database indicated that the translated amino acid sequences of the 42 047 predicted protein-encoding genes showed significant sequence similarity to the registered genes, and 12 871 were similar to TEs. Consequently, 43 266 protein-encoding genes excluding those TEs were assigned in the assembled genomic sequences, ∼72% of which showed sequence similarity to registered genes (Supplementary Table S6). It is possible that the gene number was overestimated due to fragmentation of the assembled sequences and the heterozygotic nature of the genome.

To estimate the gene coverage of the assembled genomic sequences and the accuracy of our gene prediction, we mapped core eukaryotic genes onto the assembled genomic sequences. Of the 248 core eukaryotic genes, 238 (96%) matched the entire coding regions, which increased to 242 (98%) if partial matches were included.

### 3.3. Comparison of carnation gene repertoire with those of other plant species

To compare metabolic pathway genes, we examined the genes of *B. vulgaris, A. thaliana* and *O. sativa*. Since the genomic sequences of *B. vulgaris* are not yet available, 29 830 EST sequences of *B. vulgaris* retrieved from dbEST were assembled into 14 058 unigenes consisting of 5810 contigs and 8248 singletons (total size: 9 781 432 bp) and used for comparison. For *A. thaliana* and *O. sativa*, the complete gene sets, 35 386 and 42 136 genes, respectively, were retrieved from the TAIR10 and IRGSP 1.0 databases, respectively.

The translated amino acid sequences of 43 491 coding sequences (CDSs) including splicing variants in carnation were searched against the unigenes of *B. vulgaris* and the complete gene sets of *A. thaliana* (TAIR10) and *O. sativa* (IGRSP 1.0) using BLAST with *E*-value cutoff of 1*E*–10. The distributions of the percentage of amino acid sequence identities are listed in Supplementary Fig. S5. The degree of similarity was in order *B. vulgaris*, *A. thaliana* and *O. sativa*, which is consistent with their phylogenetic relationship.

**Figure 1.**
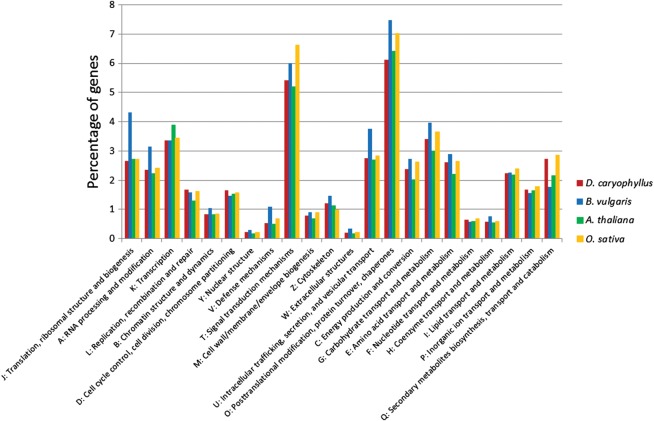
Gene assignment to KOG functional categories.

The unigenes of *B. vulgaris* and the genes of *A. thaliana* (TAIR10) and *O. sativa* (IRGSP 1.0) were classified into KOG categories (Fig. 1). The number of unigenes classified into KOG was 6937 (49.3%) in *B. vulgaris*, and the number of genes classified into KOG was 19 005 (43.7%), 18 250 (51.6%), and 18 065 (42.9%) in carnation, *A. thaliana*, and *O. sativa*, respectively. The ratio of the genes in KOG Q (Secondary metabolites biosynthesis, transport and catabolism) was relatively high in carnation. For classification of the genes based on GO slim, on the other hand, the number of unigenes classified into GO category was 5676 (40.4%) in *B. vulgaris*, and that of genes classified into GO category was 16 423 (37.8%), 21 875 (61.8%) and 20 203 (47.9%) in carnation, *A. thaliana* and *O. sativa*, respectively. This result indicated that the genes in the categories ‘nucleobase, nucleoside, nucleotide and nucleic acid metabolic process (Biological Process: BP)’, ‘cell wall (Cellular Component: CC)’ and ‘transferase activity (Molecular Function: MF)’ were relatively high in carnation (Supplementary Fig. S6).

The unigenes of *B. vulgaris* and the complete gene sets comprising 35 386 and 42 136 genes of *A. thaliana* (TAIR10) and *O. sativa* (IGRSP 1.0), respectively, were mapped onto KEGG reference pathways. As a result, 11 030 of 43 491 translated amino acid sequences of CDSs in carnation, 13 979 of 14 058 unigenes in *B. vulgaris*, 13 154 of 35 386 genes in *A. thaliana* (TAIR10) and 12 082 of 42 136 in *O. sativa* (IGRSP 1.0) were successfully mapped onto the KEGG reference pathways (Supplementary Table S7). The pathways including the genes mapped only in carnation are as follows: ‘Pentose phosphate pathway’, ‘Galactose metabolism’, ‘Ether lipid metabolism’, ‘Alanine, aspartate and glutamate metabolism’, ‘Glycine, serine and threonine metabolism’, ‘Cysteine and methionine metabolism’, ‘Metabolism of xenobiotics by cytochrome P450’ and several others.

### 3.4. Genes characteristic of carnation

#### 3.4.1. Genes for enzymes involved in phenylpropanoid biosynthetic pathways

The phenylpropanoid biosynthetic pathway that begins with phenylalanine and results in the production of anthocyanin, flavonoid and lignin is one of the most well-studied secondary metabolic pathways (Supplementary Fig. S7). Similarity searches and phylogenetic analyses^25^ for genes known to be involved in the synthesis of phenylpropanoid, flavonoid and lignin against the assembled genomic sequences of carnation was performed, and the results are summarized in Supplementary Table S8.

Similarity searches detected a single possible flavonoid 3′-hydroxylase (F3′H) homologue among 241 genes homologous to cytochrome P450 in the genome of carnation cv. ‘Francesco’. The *F3′H* gene in cv. ‘Mrs. Purple’ is known to code for an active protein of 514 amino acid residues.^4^ By contrast, the F3′H homologue in cv. ‘Francesco’ contains one extra guanidine nucleotide in the potential coding region, resulting in the production of a truncated 193 amino acid peptide.

In addition, a total of 84, 25, 120, 93 and 139 putative homologues of glutathione *S*-transferases, multidrug and toxic compound extrusion (MATE)-type transporter, UDP-sugar dependent glycosyltransferase, Myb transcription factors and bHLH transcription factors, which are known to be involved in the phenylpropanoid biosynthetic pathway, were assigned in the assembled genomic sequences. Details of these genes as well as the results of phylogenetic analysis are shown in Supplementary Figs S8–S12.

#### 3.4.2. Genes involved in betalain synthesis

Betalains are commonly synthesized instead of anthocyanin in the order Caryophyllales, except for two families, Caryophyllaceae and Molluginaceae. Although carnation is classified under the family Caryophillaceae and produces anthocyanin as the main plant pigment in petals, and often in other organs, carnation may also contain the genes required to produce betalains. Three characteristic enzymes are involved in synthesis of betalains, i.e. l-dihydroxyphenylalanine (DOPA) 4,5-dioxygenase (DOD),^6,26^ cytochrome P450 monooxygenase (CYP76AD)^27^ and *cyclo*-DOPA glucosyltransferase (*c*D5GT), in the UDP-dependent glycosyltransferase (UGT) family.^28,29^

One copy of a DOD homologue (*Dca8668*) was found in the carnation genomic sequences by similarity searching. Multiple alignments revealed that the amino acid sequence of the carnation DOD homologue contains the conserved motif typical of non-betalain-producing plants (Supplementary Fig. S13). This result suggests that the carnation DOD homologue does not catalyze the formation of betalamic acid.

CYP76AD belongs to the cytochrome P450 monooxygenase (CYP) family, which is one of the most divergent families in higher plants,^27^ with 241 homologues of CYP detected in the carnation genomic sequences. Dca32662 showed the highest amino acid identity (66.4%) with that of *B. vulgaris* CTP76AD1. However, the fact that this protein belongs to CYP76C implied that carnation genome does not encode betalain-related CYP family protein. A single homologue of *Mirabilis jalapa cD5GT* (*MjcD5GT*) gene was found. The deduced amino acid sequence of this UGT homolog showed 58.4% identity to MjcD5GT and 62.5% to *Celosia cristata* cD5GT.

#### 3.4.3. Genes involved in chlorophyll and carotenoid synthesis

Similarity searches against the assembled genomic sequences in carnation identified all of the genes involved in the chlorophyll metabolic pathway in the carnation genome (Supplementary Table S9). We found multiple genes encoding putative isozymes, including glutamate-1-semialdehyde 2,1-aminotransferase (*GSA*), 5-aminolevulinate dehydrogenase (*HEMB*), porphobilinogen deaminase (*HEMC*), uroporphyrinogen III decarboxylase (*HEME*), magnesium chelatase D subunit (*CHLD*)*,* Mg-protoporphyrin IX methyltransferase (*CHLM*), Mg-protoporphyrin IX monomethylester cyclase (*CRD*), protochlorophyllide oxidoreductase (*PORA*) and divinyl chlorophyllide *a* 8-vinyl reductase (*DVR*), which are involved in chlorophyll synthesis in carnation. By contrast, all of the enzymes involved in the chlorophyll cycle and chlorophyll degradation are likely to be encoded by a single gene. STAY-GREEN (*SGR*), a protein involved in senescence-related chlorophyll degradation, is encoded by three genes.

Similarity searches revealed that most of the enzymes involved in carotenoid biosynthesis are encoded by single genes in the carnation genome (Supplementary Table S10). In addition, two homologues of 9-*cis*-epoxycarotenoid dioxygenase (*NCED*) genes involved in abscisic acid biosynthesis in *A. thaliana*,^30^ two homologs of carotenoid cleavage dioxygenase (*CCD4*) genes as well as single homologues each of *CCD1*, *CCD7* and *CCD8*, genes related to different enzyme activities and substrate specificities,^30^ were assigned (Supplementary Fig. S14). No ESTs encoding CCDs were found in the carnation EST dataset (Supplementary Table S10),^13,31^ suggesting that their expression levels are low.

#### 3.4.4. Disease resistance genes

We searched for nucleotide-binding site-leucine-rich repeat (NBS-LRR) genes in the assembled genomic sequences of carnation and assigned 217 NBS-containing potential Resistance (*R*) genes (Supplementary Table S11). The number of NBS-LRR genes in carnation is larger than that of cucumber,^32^ melon^33^ and papaya,^34^ but smaller than that of tomato,^35^ grape^36^ and rice.^37^ In this study, only three potential genes with Toll/interleukin-1 receptor (TIR) domains at the N-terminal were identified, two of which lack LRRs, while 69 genes containing a coiled-coil motif were assigned, 52 of which lack LRRs. This result is consistent with the previous observation that the genome of *B. vulgaris* lacks TIR-type resistance genes despite the fact that this plant is a dicot.^38^ Our present results support the assumption that the loss of TIR-type resistance genes is not restricted to cereals or monocots in general,^38^ indicating the unique feature of *R* gene evolution in Caryophyllales. It should also be noted that more than 50% of the 114 NBS-containing genes lack N- and C-terminal domains (Supplementary Table S11), with 30 genes (NL) possessing only NBS-LRR domains.

The 217 potential NBS-type *R* genes predicted in this study were assigned to 125 scaffolds. Of the 125 scaffolds, 87 contain single NBS genes, while the other scaffolds contain multiple NBS genes. The positions of eight scaffolds containing five or more NBS genes are shown in Supplementary Fig. S15. Approximately 30% of the NBS-type *R* genes are clustered in these scaffolds, and the scaffold most abundant in NBS-type *R* genes is No.146, which contains 11 genes. These results suggest that *R* genes are unevenly distributed in the carnation genome, as was reported for a wide range of plants.^33–35,37^

#### 3.4.5. Genes involved in ethylene metabolism

Carnation has been used as a model system to study the mechanism of ethylene-induced flower senescence.^10^ Components of the ethylene signal transduction pathway, such as the ethylene receptors, Constitutive Triple Response (CTR), Ethylene-Insensitive 2 (EIN2) and EIN3, regulate a series of senescence-related genes.^10^ By searching the carnation genomic sequences, six putative genes for ethylene receptors (DcETR1, DcETR2, DcETR3, DcETR4, DcERS1 and DcERS2), two genes for CTR, two genes for EIN2 and three EIN3-like genes were identified (Supplementary Table S12). Phylogenetic analysis classified six ethylene receptors in carnation into two subfamilies; DcETR1, DcERS1 and DcERS2 are in Subfamily 1, which contains conserved histidine kinase domains, and DcETR2, DcETR3 and DcETR4 are in Subfamily 2 (Supplementary Fig. S16).

With respect to ethylene biosynthesis, three 1-aminocyclopropane-1-carboxylic acid (ACC) synthase genes (*DcACS1*, *DcACS2* and *DcACS3*) and one ACC oxidase gene (*DcACO1*) have been identified.^39^ Based on sequence similarity, we found six more genes for ACS (*DcACS*4 –*DcACS9*) and four more genes for ACO (*DcACO2*–*DcACO5*; Supplementary Table S12). Notably, the putative gene products of DcACS4, DcACS5 and DcACS6 lack the motifs BOX6 and BOX7, BOX1 and BOX2, and BOX6, respectively, while those of other ACSs contain all seven conserved BOXs found in ACS isozymes in *A. thaliana*.^40^ Since only eight out of 12 ACS genes are catalytically active in *A. thaliana*,^40^ it is probable that all of the *ACS* identified in the carnation genome do not encode active ACS for synthesizing ethylene.

#### 3.4.6. Genes involved in carbohydrate metabolism and cell wall modification during flower opening

Large amounts of soluble carbohydrate are required for flower opening as substrates for respiration and cell wall synthesis. Accumulation of substantial amounts of pinitol, one of the rare sugars potentially involved in salinity tolerance, is a unique aspect of sugar metabolism during flower opening in carnation.^41^ One gene (*Dca24344*) showing strong homology to known genes encoding *myo*-inositol methyl transferase (IMT), which catalyzes the conversion of *myo*-inositol to pinitol, was found in the carnation genome.

Xyloglucan endotransglucosylase/hydrolase (XTH), which is involved in the hydrolysis and reconstruction of xyloglucan in the matrix polysaccharides of the cell wall,^42^ is believed to play an essential role in increasing cell wall extensibility followed by water uptake during the process of petal cell expansion.^43^ Genome-wide analysis has revealed 33 and 29 genes in the XTH family in *A. thaliana*^44^ and *O. sativa*,^45^ respectively. In the carnation genomic sequences, 32 genes putatively encoding XTH were detected (Supplementary Table S13 and Figs. S17, S18), 11 of which were reported to be expressed in flowers and in some vegetative tissues.^13,46^

#### 3.4.7. Genes related to floral scent

Floral scent is a notable breeding target for carnation.^47^ Methylation by *S*-adenosyl-l-methionine-dependent methyltransferases belonging to SABATH family,^48^ is an important catalytic process for emission of scent components from flowers. Methyl benzoate that is a major scent component of modern carnation cultivars,^47^ is also derived from the methylation of benzoic acid. A similarity search against the carnation genomic sequences detected 11 genes in the SABATH family (*DcSABATH1-11*), which are candidate genes of benzoic acid methyltransferase in carnation (Supplementary Table S14). Phylogenetic analysis of functionally characterized members of SABATH family showed that benzoic acid and salicylic acid methyltransferase form a monophyletic lineage irrespective of plant species (Supplementary Fig. S19).^49^ In contrast, DcSABATHs, SABATH members in carnation, did not strongly associate with this lineage.

### 3.5. Database and data retrieval

All of the information about the assembled scaffold sequences, known and novel repetitive sequences, genes for non-coding RNA (tRNA, rRNA, snoRNA and miRNA) and potential protein-encoding genes is available through the Carnation DB (http://carnation.kazusa.or.jp).

All the sequence data obtained in this study is available under the BioProject ID, PRJDB1491. The accession number of the reads sequenced by Illumina HiSeq 1000 are as follows: PE (insert size = 500 bp): DRX012625, OF (insert size = 180 bp): DRX012624, MP (insert size = 3 kb): DRX012626, MP (insert size = 5 kb): DRX012627. The accession numbers of the reads sequenced by Roche GS FLX+ are as follows: SE: DRX012628, PE (insert size = 4 kb): DRX012629. The nucleotide sequences of assembled scaffolds can be retrieved under the accession numbers DF340864-DF357213 (16 350 entries).

## Supplementary Data

Supplementary Data are available at www.dnaresearch.oxfordjournals.org.

## Funding

This work was funded by grants from the Yoshio Itoh Fund of Tokyo University of Agriculture and Technology, and the Kazusa DNA Research Institute Foundation. Construction of the BAC library was supported by a genome-support grant from the National Institute of Agrobiological Sciences (NIAS) and JSPS KAKENHI grant number 24780038.

## Supplementary Material

Supplementary Data
