# Supplementary material for: Sequence Analysis of the Genome of Carnation (Dianthus caryophyllus L.)
Source: DNA Res. 2013 Dec 17;21(3):231–41. doi: 10.1093/dnares/dst053 (PMC4060945; doi:10.1093/dnares/dst053)
Supplement: Supplementary Data [file supp_21_3_231__index.html]

Sequence Analysis of the Genome of Carnation (Dianthus caryophyllus L.) — Supplementary Data 

# Sequence Analysis of the Genome of Carnation (*Dianthus caryophyllus* L.)

## Supplementary Data

Supplementary Data

**Files in this Data Supplement:**

- Supplementary Figures - pdf file
- Supplementary Tables - xlsx file
