# Supplementary material for: Sequence Analysis of the Genome of Carnation (Dianthus caryophyllus L.)
Source: DNA Res. 2013 Dec 17;21(3):231–41. doi: 10.1093/dnares/dst053 (PMC4060945; doi:10.1093/dnares/dst053)
Supplement: Supplementary Data [file supp_dst053_dst053supp_figures.pdf]

Assembly data	Assembler	Read library	N50 (Kb) [a]	Total length (Mb) [a]
(1) 454-contigs	Newbler	SE, 4-kb PE	2.6	384
(2) SOAP-contigs	SOAPdenovo2	500-bp PE	1.6	283
(3) FERMI-contigs	FERMI	500-bp PE	2.1	334
(4) ALLPATHS-scafs	ALLPATHS-LG	180-bp OF, 500-bp PE, 3-Kb MP, 5-Kb MP	69.7	369

[a] values for assemblies with ≥ 500 bp

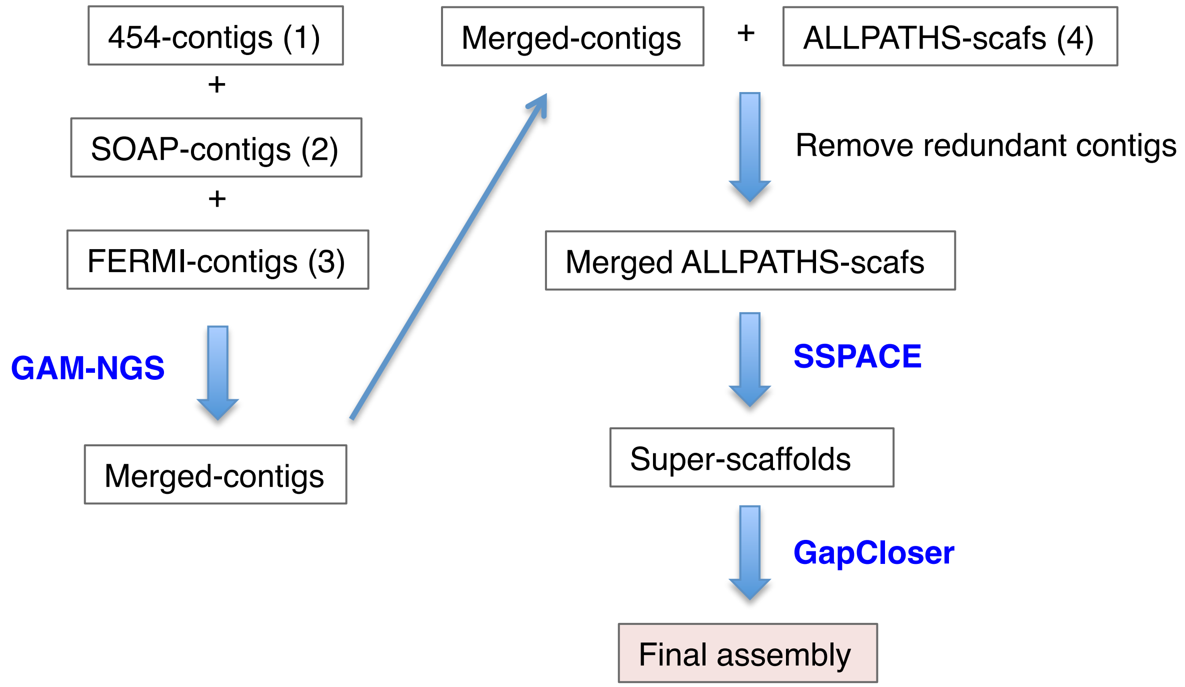

Supplementary Figure S1. Strategy for sequencing and data assembly.

Sequence data obtained by the HiSeq 1000 sequencer were subjected to assembly using the ALLPATHS-LG program. In parallel, 3' low-quality regions of the HiSeq sequence data from the PE and MP libraries were trimmed with FASTX-toolkit (http://hannonlab.cshl.edu/fastx_toolkit/) and used for assembly with SOAPdenovo2 and FERMI. Sequence data from the Roche 454 obtained from the SE and PE libraries were assembled by Newbler 2.7. The three contig sets generated with Newbler, SOAPdenovo2, and FERMI were sequentially merged with a GAM-NGS merger, and the merged contig set was combined with the ALLPATH-scaffold set, followed by removal of redundant contigs. The merged assemblies were further scaffolded with SSPACE and gapclosed with GapCloser to generate a final assembly set.

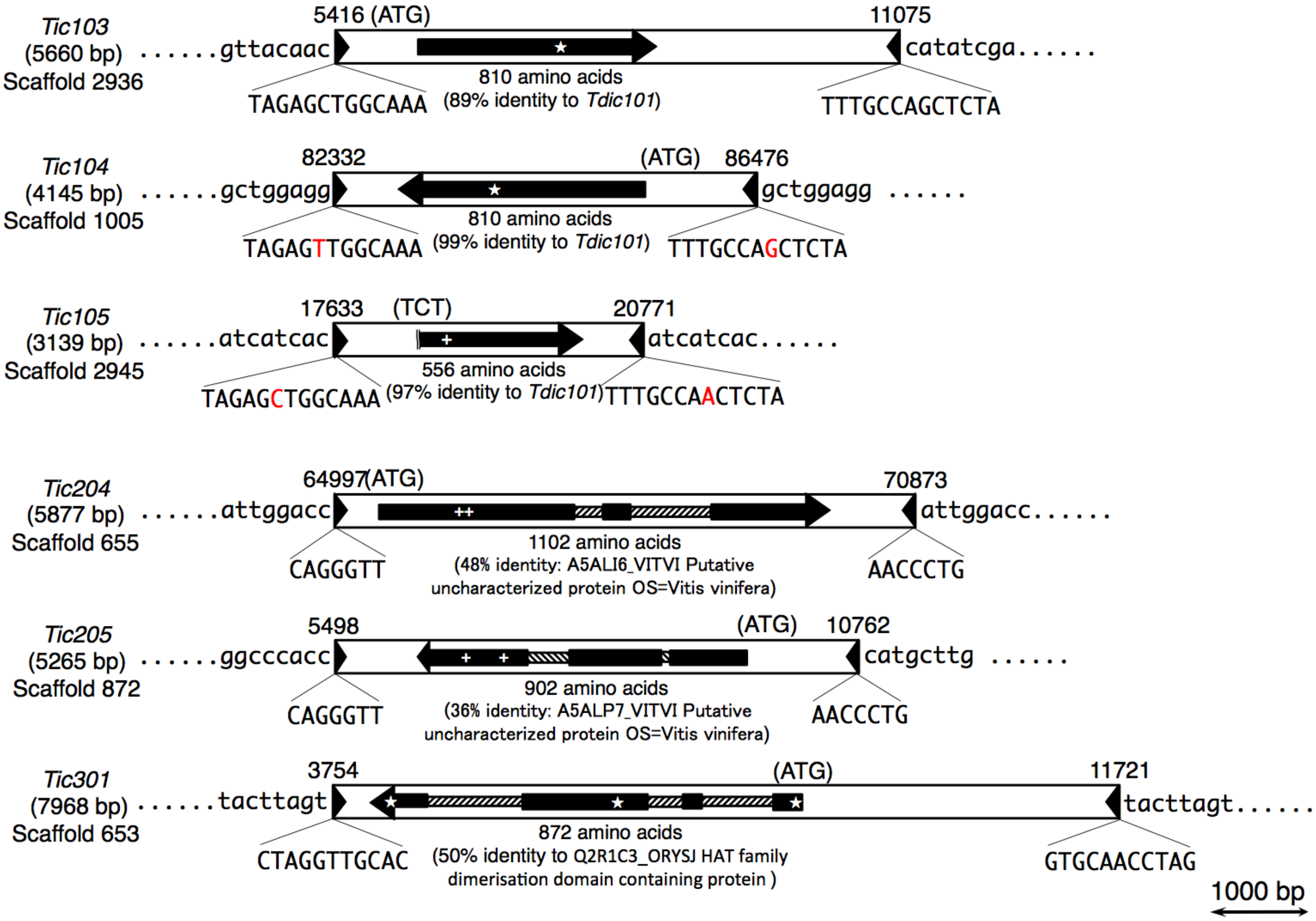

Supplementary Figure S2. Structure of members of the *hAT* (*Ac*/*Ds*) element.

Tic101, a member of *hAT* (*Ac*/*Ds*) elements, was identified as an autonomous transposable element encoding an active transposase protein.[a] An intensive search for the *Tic101* sequence against the assembled genomic sequences detected only a single homolog, designated *Tic104*, whose sequence is 99% identical (Supplementary Table S4) to that of *Tdic101*.[a] *Tic104* has one nucleotide substitution in a terminal-inverted repeat sequence and another in the coding region of the transposase gene to generate a stop codon, which may have led to inactivation of transposition of this element.[b] Black boxes indicate putative open reading frames (ORFs). White crosses indicate positions of a stop codon. White stars show positions of a nucleotide insertion or deletion to disrupt ORF sequence.

a. Momose, M., Nakayama, M., Itoh, Y., Umemoto, N., Toguri, T. and Ozeki, Y. 2013, An active *hAT* transposable element causing bud mutation of carnation by insertion into the flavonoid 3'-hydroxylase gene, *Mol. Genet. Genomics*, **288**, 175-184.
b. Itoh, Y., Hasebe, M., Davies, E., Takeda, J. and Ozeki, Y. 2003, Survival of *Tdc* transposable elements of the *En/Spm* superfamily in the carrot genome, *Mol. Gen. Genomics*, **269**, 49-59.

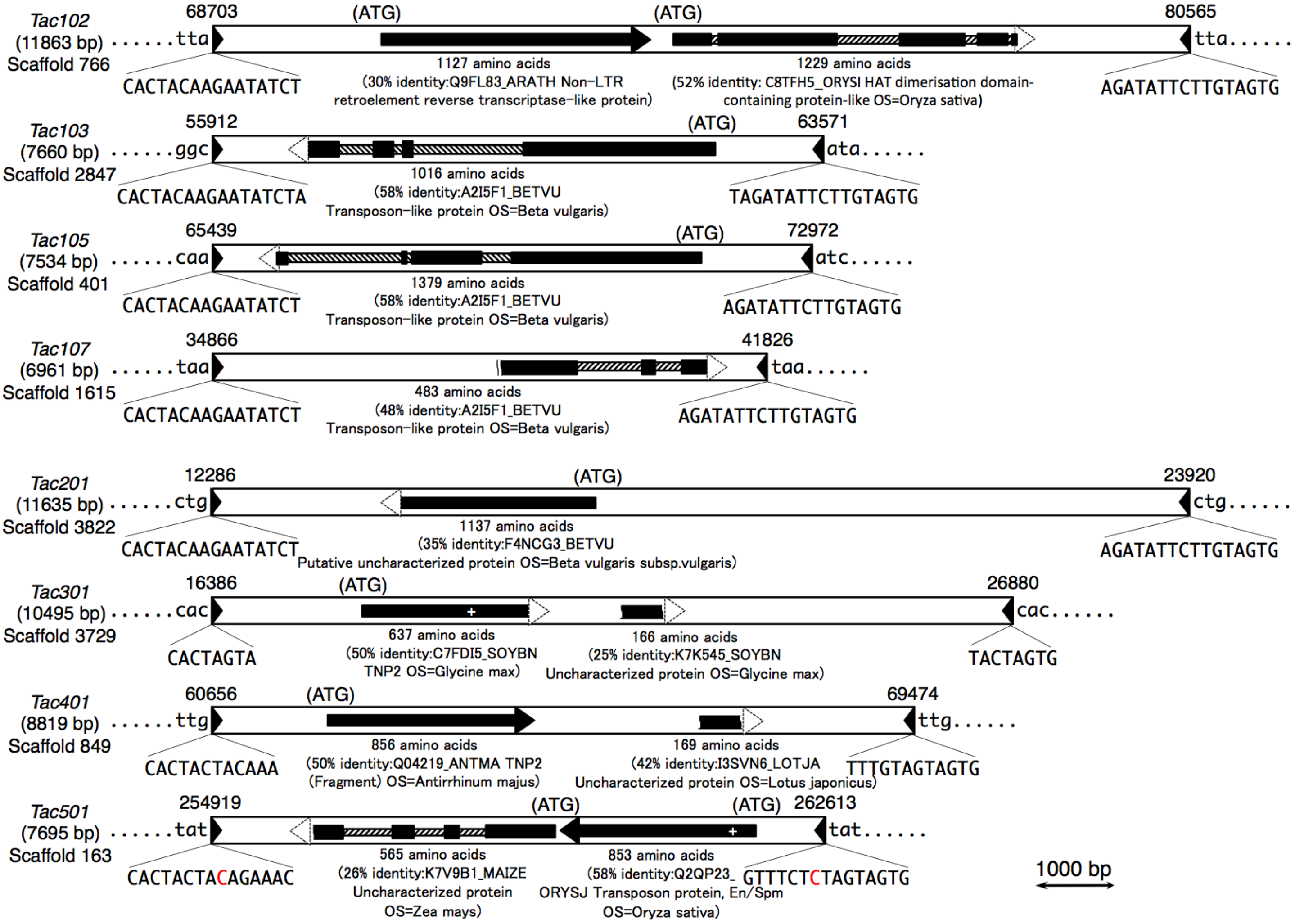

Supplementary Figure S3. Structure of members of the CACTA element.

The CACTA element (*En*/*Spm*), *dTac1* was first identified in a gene for glutathione-*S*-transferase, here designated *Tac101*, which is not likely to encode notable proteins for transposases.[a] *In silco* screening of the genomic sequences of 'Francesco' failed to identify its autonomous elements or intact sequences of the transposases TNP1 and TNP2. Since transposition of *Tac101* is responsible for variegated color deepness in the petals of 'Daisy-VP',[a] intact gene(s) for active transposases should exist in 'Daisy-VP' and in other carnations, but not in 'Francesco'. A similarity search against the 'Francesco' genomic sequences detected other types of CACTA elements, designated *Tac201*, *Tac301*, *Tac401* and *Tac501* (Supplementary Table S4). None of these is likely to encode intact transposases that are active for transposition. Black boxes indicate putative open reading frames (ORFs). White crosses indicate positions of a stop codon.

a. Sasaki, N., Nishizaki, Y., Uchida, Y., et al. 2012, Identification of the *glutathione* S-*transferase* gene responsible for flower color intensity in carnations. *Plant Biotechnol*., **29**, 223-227.

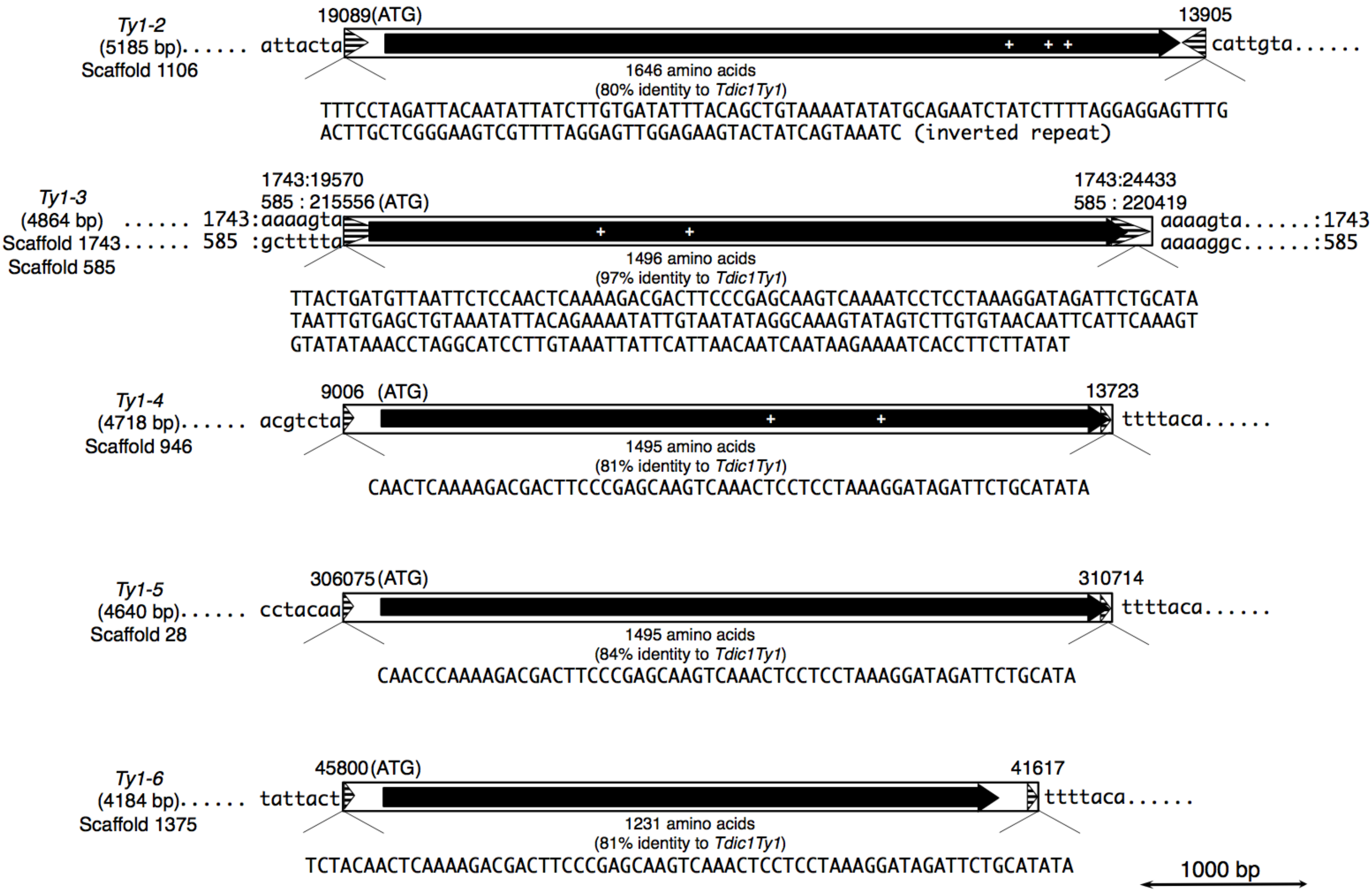

Supplementary Figure S4. Structure of members of the *Ty1* family.

The genome of 'Francesco' contains an *acyl-glucose-dependent anthocyanin 5-glucosyltransferase* (*AA5GT*) gene with an insertion by *Ty1-1*(*Ty1dic1*), resulting in synthesis and accumulation of pelargonidin 3-*O*-malylglucoside that lacks its glucose moiety at the 5 position. *Ty1-1* is inserted at the same position of the *AA5GT* gene in the 'Francesco' genome as the *Ty1dic1* insertion in *AA5GT* in the 'Xeria' and 'Master' genomes,[a] but *Ty1-1* has one nucleotide substitution in the coding region of the transposase gene to generate a stop codon different from *Ty1dic1*; *Ty1-1* can no longer move by the copy-and-paste mechanism. Similarity searching of the assembled genomic sequences indicated that there were six genes for *Ty1-1* longer than 1 kb (Supplementary Table S4), which is consistent with the observation that only four to five notable bands were detected by Southern blotting. [a] Black boxes indicate putative open reading frames (ORFs). White crosses indicate positions of a stop codon.

a. Nishizaki, Y., Matsuba, Y., Okamoto, E., Okamura, M., Ozeki, Y. and Sasaki, N. 2011, Structure of the acyl-glucose-dependent anthocyanin 5-*O*-glucosyltransferase gene in carnations and its disruption by transposable elements in some varieties. *Mol. Gen. Genomics*, **286**, 383-394.

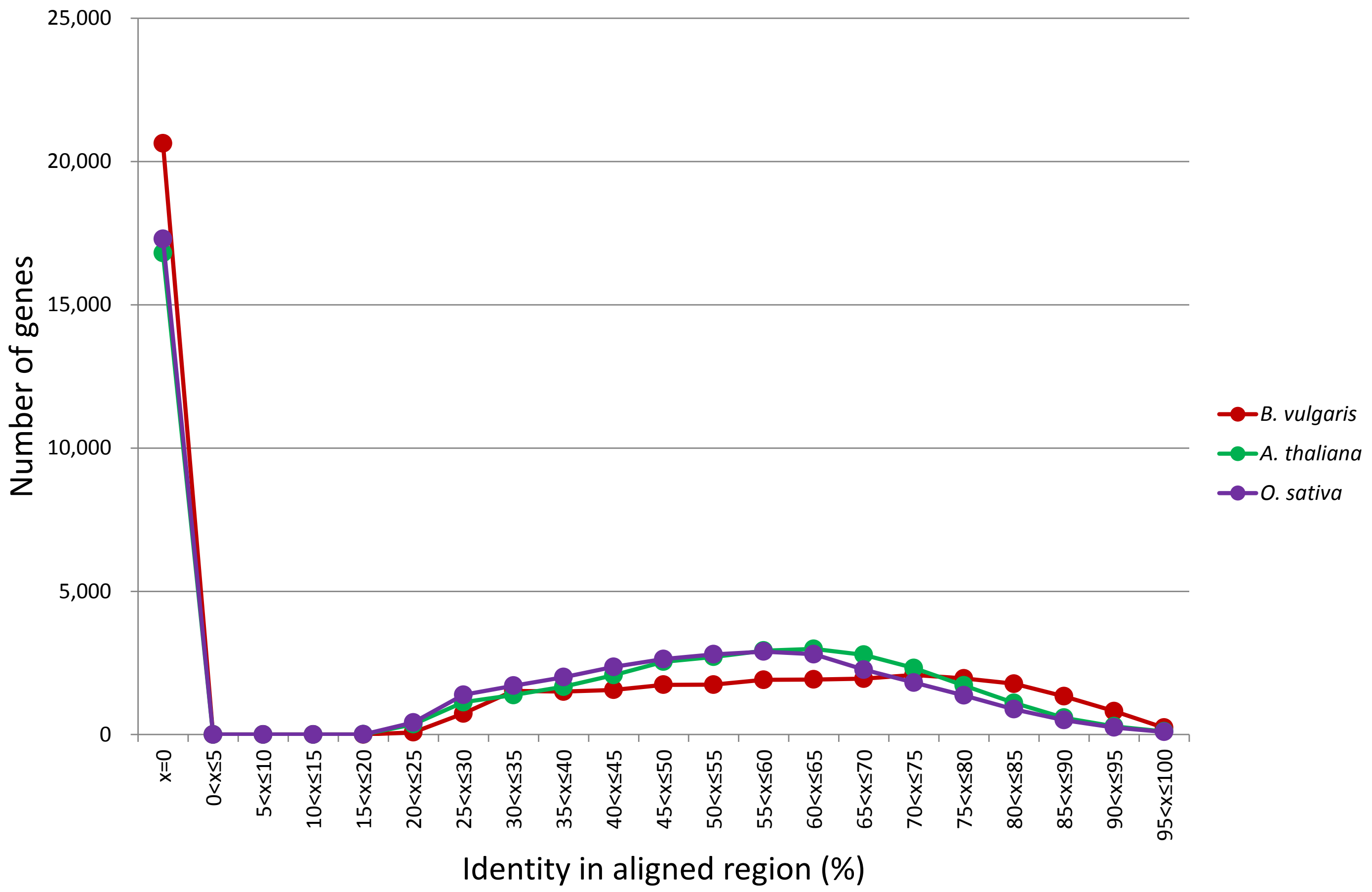

Supplementary Figure S5. Distribution of percent identity in amino acid sequences of three plant species.

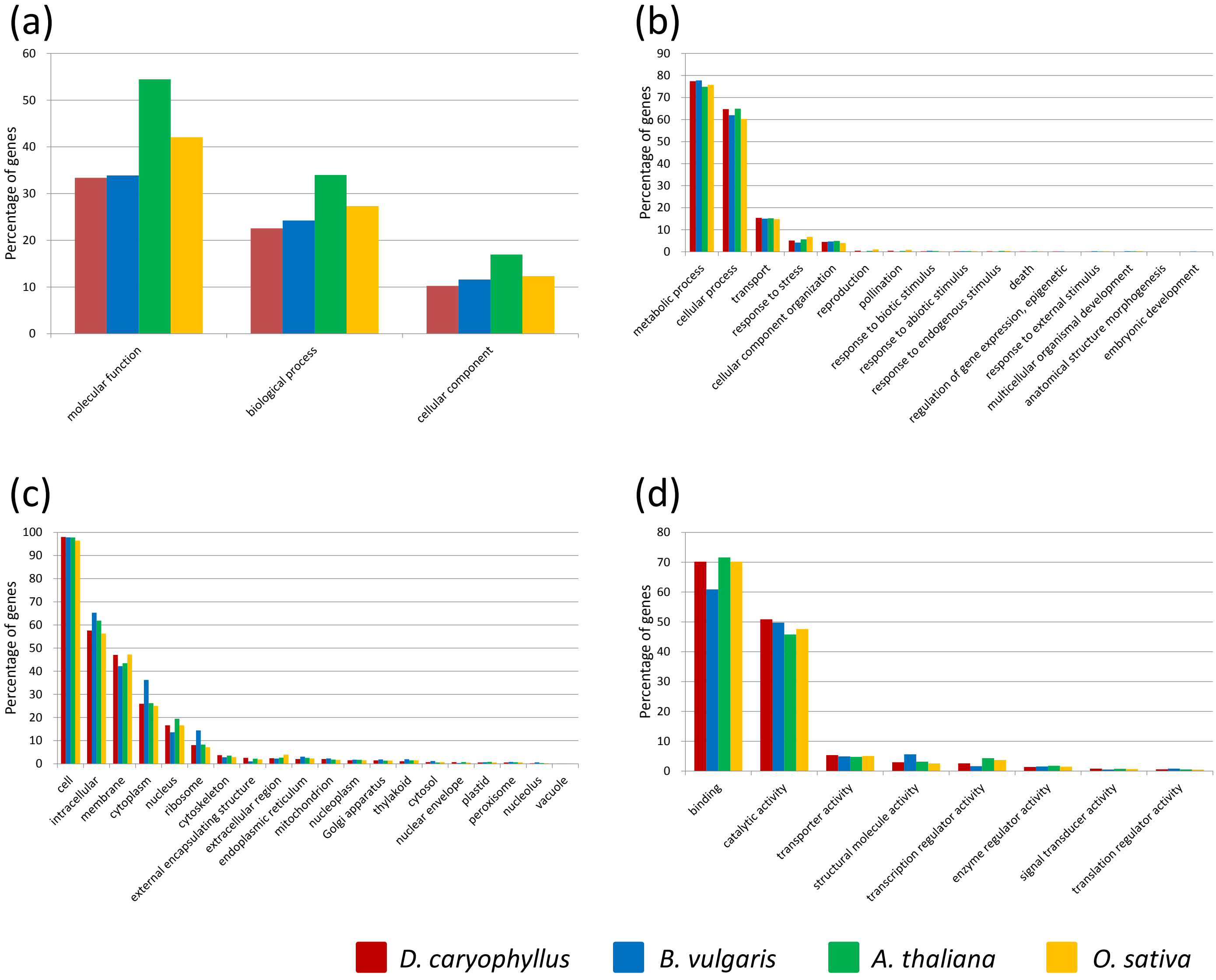

Supplementary Figure S6. Distribution of putative genes in GO categories.

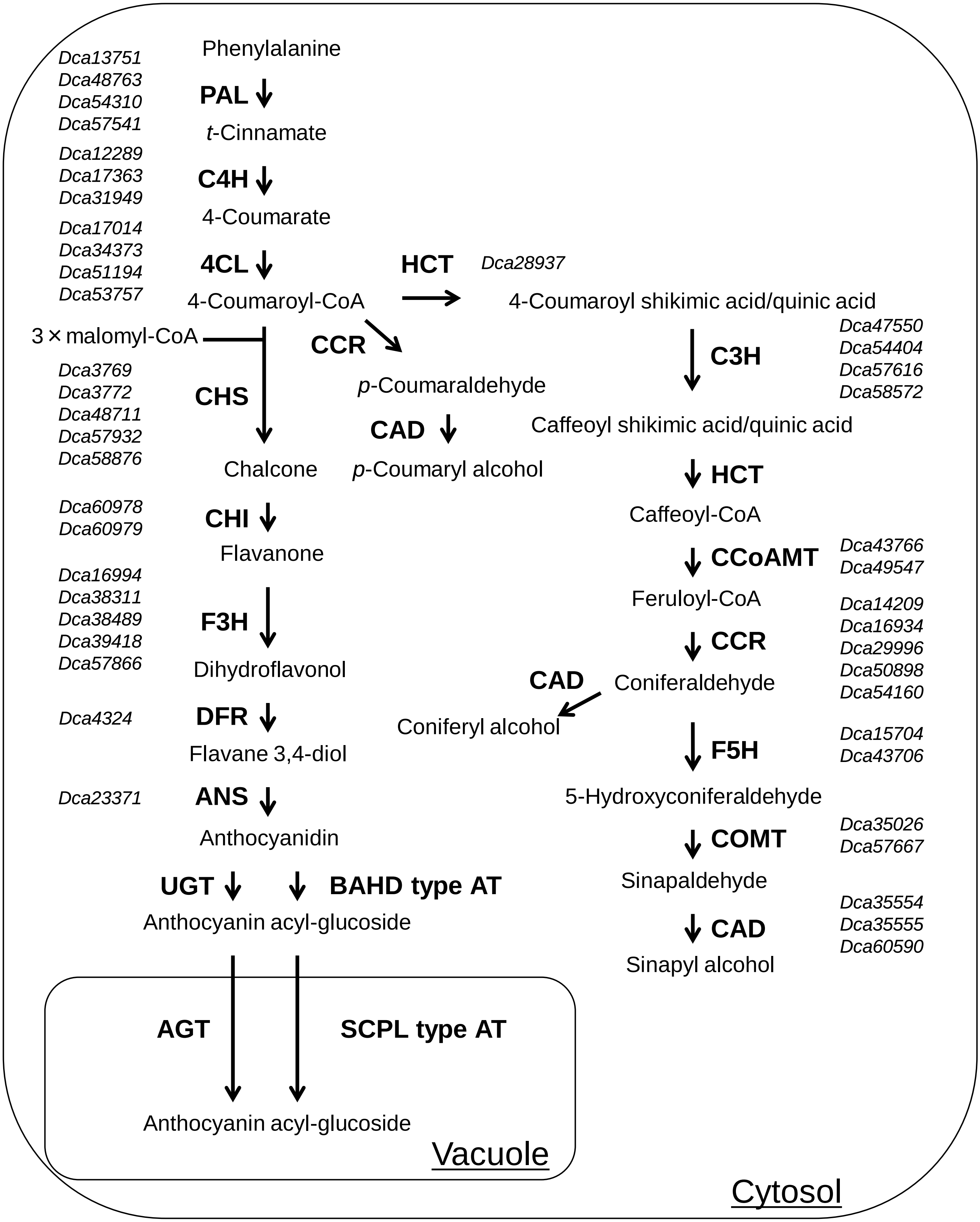

Supplementary Figure S7. Phenylpropanoid biosynthetic pathway in carnation.

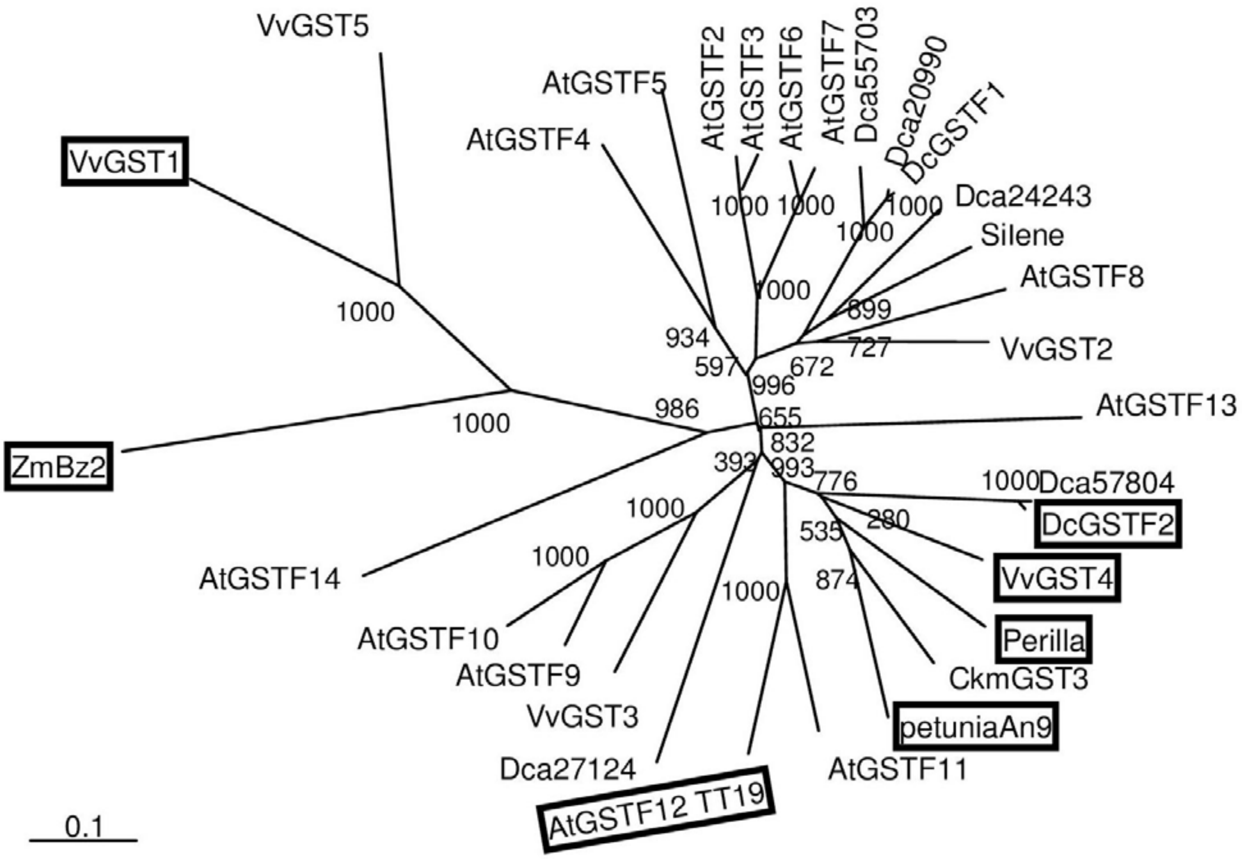

Supplementary Figure S8. Phylogenetic analysis of glutathione *S*-transferases.

GSTs in plants form a large and diverse group catalyzing the conjugation of various compounds with glutathione that play important roles in the sequestration of secondary metabolites and xenobiotics.[a] A total of 84 potential genes for GST were identified in the carnation genomic sequences. Dca57804 and Dca20990, both of which play important roles in anthocyanin transport, showed 98% amino acid identity to DcGSTF2 and DcGSTF1, respectively.[b]

Amino acid sequences were aligned with CLUSTALW (available at DNA Data Bank of Japan, http://clustalw.ddbj.nig.ac.jp/top-e.html), and a phylogenetic tree was generated by the TreeView program[c] Numbers next to the nodes are bootstrap values from 1000 replications . The bar indicates a genetic distance of 0.1. The deduced amino acid sequences were retrieved from the nucleotide sequences of the DDBJ/EMBL/GenBank databases. Glutathione *S*-transferases that have been experimentally shown to be involved in anthocyanin transport are boxed. *Dianthus caryophyllus* (DcGSTF1, AB688110; DcGSTF2, AB688111), *A. thaliana* (AtGSTF2, At4g02520; AtGSTF3, At2g02930; AtGSTF4, At1g02950; AtGSTF5, At1g02940; AtGSTF6, At1g02930; AtGSTF7, At1g02920; AtGSTF8, At2g47730; AtGSTF9, At2g30860; AtGSTF10, At2g30870; AtGSTF11, At3g03190; AtGSTF12, At5g17220; AtGSTF13, At3g62760; AtGSTF14, At1g49860), *Vitis vinifera* (VvGST1, AY156048; VvGST2, EF088687; VvGST3, EF469244; VvGST4, AY971515; VvGST5, EF140721), *Silene cucubalus* (Silene, M84968), cyclamen hybrid (CkmGST3, AB682678), *Petunia hybrida* (petunia An9, Y07721), *Perilla frutescens* (Perilla, AB362191) and *Zea mays* (ZmBz2, U14599).

a. Dixon, D.P., Skipsey, M. and Edwards, R. 2010, Roles for glutathione transferases in plant secondary metabolism, *Phytochemistry*, **71**, 338–350.
b. Sasaki, N., Nishizaki, Y., Uchida, Y., et al. 2012, Identification of the *glutathione* S-*transferase* gene responsible for flower color intensity in carnations, *Plant Biotechnol*., **29**, 223-227.
c. Page, R.D., et al. 1996, TreeView: an application to display phylogenetic trees on personal computers, *Comput. Appl. Biosci.*, **12**, 357-358.

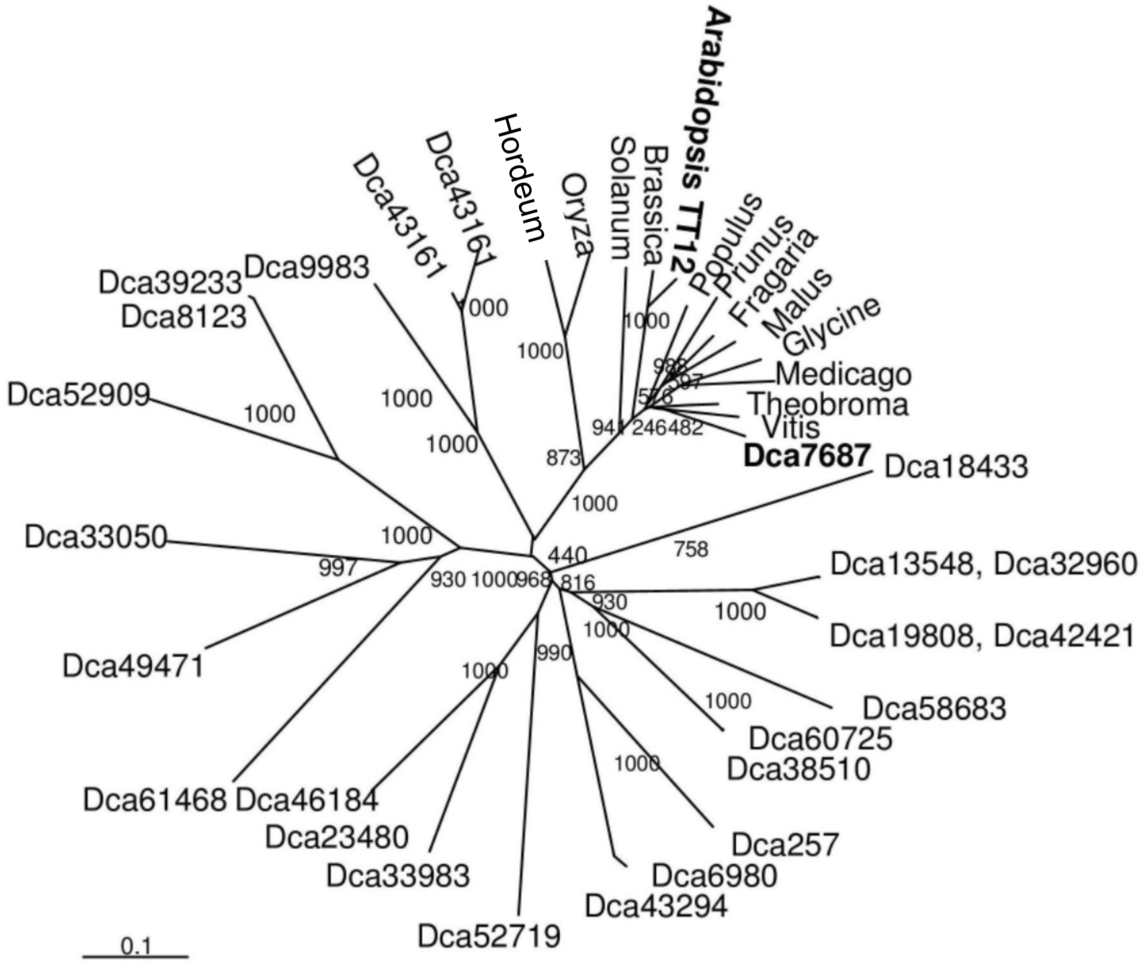

Supplementary Figure S9. Phylogenetic analysis of MATE-type transporter proteins.

The *TT12* gene, encoding a MATE-type transporter in *A. thaliana*, transports proanthocyanidins,[a] glycosylated flavan-3-ols[b] and cyanidin 3-glucoside.[c] We found 25 genes homologous to TT12 in *A. thaliana* and other plants in the genomic sequences of carnation. Phylogenetic analysis indicated that Dca7687 or Dca18433, showing 69% amino acid identity with the Arabidopsis TT12 product, may be a corresponding ortholog in carnation.

Twenty-five MATE proteins in carnation that are most homologous to Arabidopsis TT12 and homologs from other plants were included. The analysis was carried out as described in the legend of Supplementary Figure S8. Genes subjected to this analysis were as follows: *Brassica rapa* (Brassica, ACJ36214), *Theobroma cacao* (Theobroma, EOX93053), *Fragaria vesca* (Fragaria, XP_004290839), *Malus x domestica* (Malus, ADO22712), *Vitis vinifera* (Vitis, CBI22062), *Populus trichocarpa* (Populus, XP_002307572), *Prunus persica* (Prunus, EMJ16452), *Glycine max* (Glycine, XP_003535155), *Solanum lycopersicum* (Solanum, XP_004252047), *Medicago truncatula* (Medicago, ACX37118), *Hordeum vulgare* (Hordeum, BAK03595) and *Oryza sativa* (Oryza, EAY83897).

a. Debeaujon, I., Peeters, A.J.M., Léon-Kloosterziel, K.M. and Koornneef, M. 2001, The TRANSPARENT *TESTA12* gene of Arabidopsis encodes a multidrug secondary transporter-like protein required for flavonoid sequestration in vacuoles of the seed coat endothelium, *Plant Cell,* **13**, 852-871.

b. Marinova, K., Pourcel, L., Weder, B., et al. 2007, The Arabidopsis MATE transporter TT12 acts as a vacuolar flavonoid/H^+-antiporter active in proanthocyanidin-accumulating cells of the seed coat, *Plant Cell*, **19**, 2023-2038.

c. Zhao, J. and Dixon, R.A. 2009, MATE transporters facilitate vacuolar uptake of epicatechin 3′-O-glucoside for proanthocyanidin biosynthesis in *Medicago truncatula* and Arabidopsis, *Plant Cell*, **21**, 2323-2340.

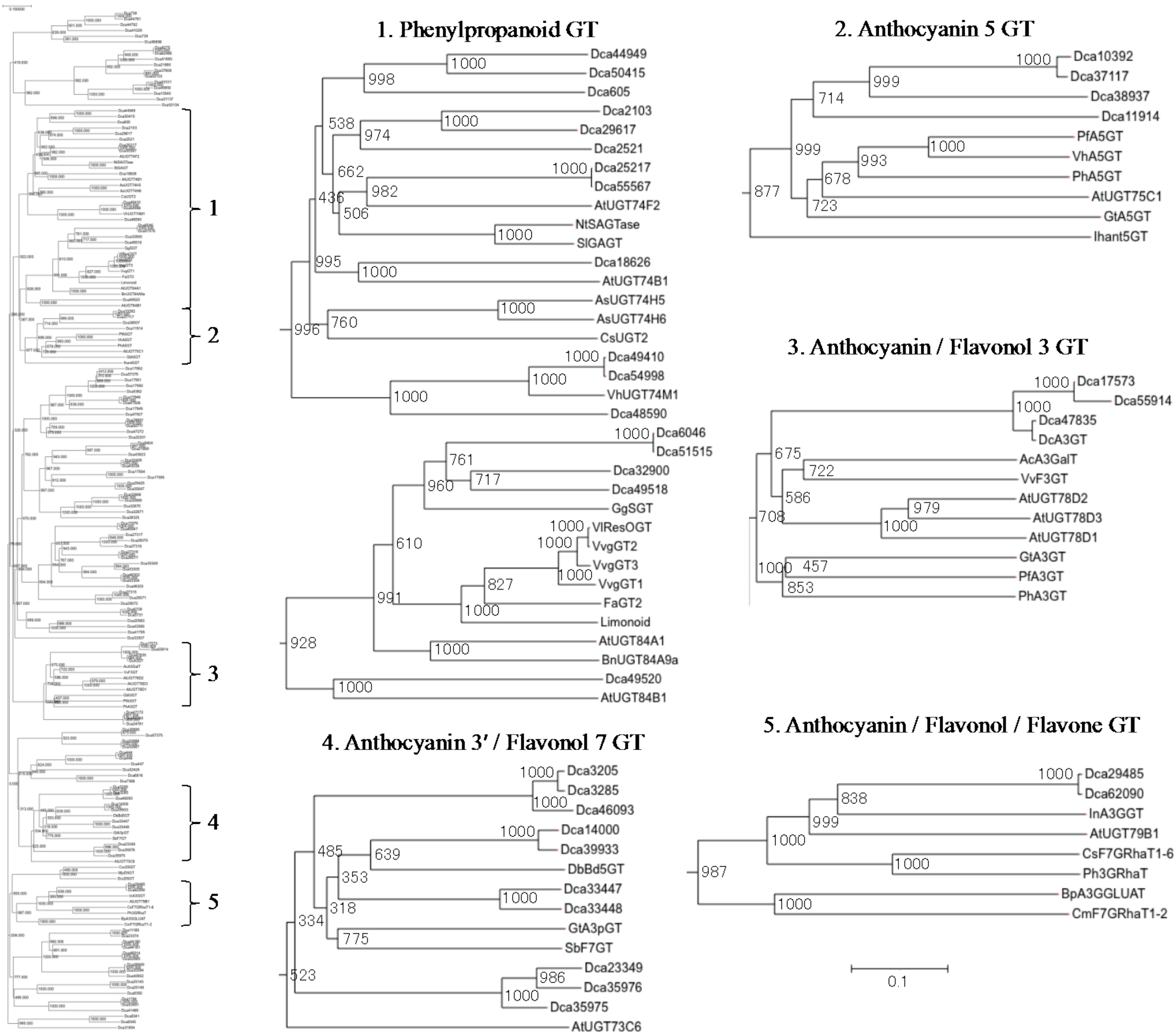

Supplementary Figure S10. Phylogenetic analysis of UDP-sugar dependent glycosyltransferase (UGT)

In the cytosol, glycosylation of flavonoids is catalyzed by UGT. A total of 120 genes showing sequence similarity to genes for UGTs were found in carnation, which is comparable to *A. thaliana* (120 genes).[a] Phylogenetic analysis showed that four genes, Dca10392, Dca11914, Dca37117 and Dca38937, were classified into Anthocyanin 5GT. Since glucosylation of anthocyanin is catalyzed by AA5GT in carnation, these genes may be involved in the glycosylation of other flavonoids.

a. Yonekura-Sakakibara, K. and Hanada, K. 2011, An evolutionary view of functional diversity in family 1 glycosyltransferases, *Plant J.*, **66**, 182-193.

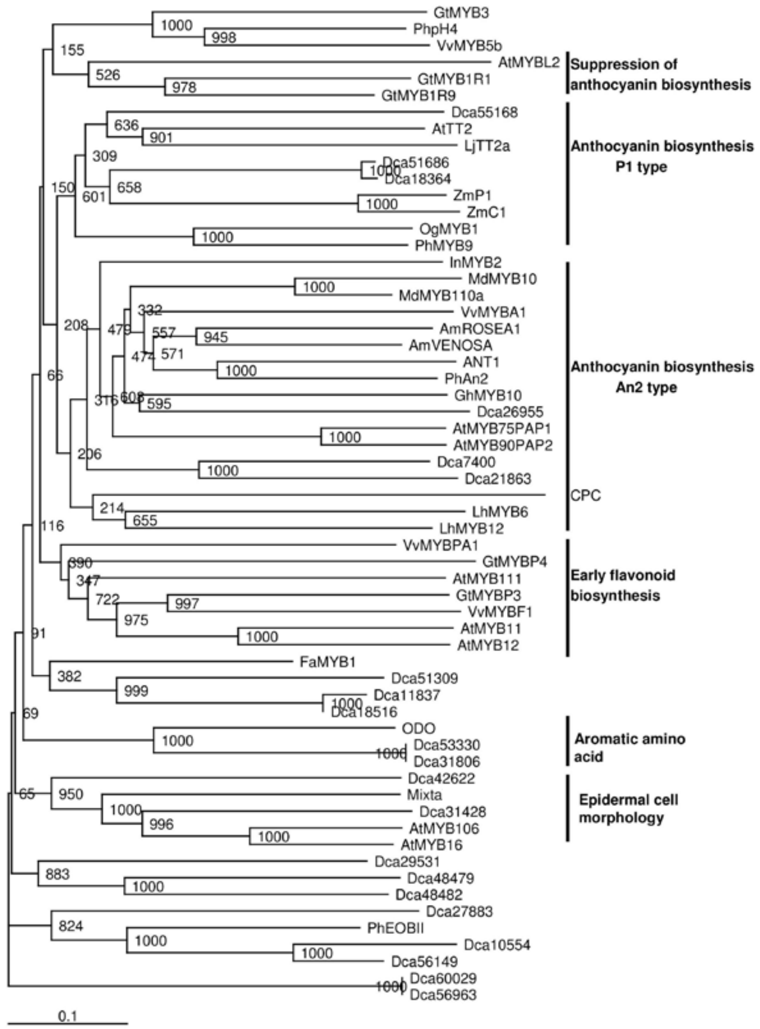

Supplementary Figure S11. Phylogenetic analysis of Myb transcription factors.

Myb transcription factors (TFs) are mostly diverse and determine the expression of a subset of flavonoid biosynthetic genes.[a] A total of 93 genes were deduced to be potential genes for Myb TFs in carnation, while 100 or more such genes were identified in the genome of *A. thaliana*. R2R3-MYB-TFs. These genes function in the regulation of anthocyanin biosynthesis and are divided into two subgroups, *AN2* and *C1*.[b] Dca26955, which belongs to the *An2* subgroup, may be involved in the regulation of anthocyanin biosynthesis, while Dca55168, Dca51686 and Dca18364 (classified in the *C1* subgroup) may regulate proanthocyanidin biosynthesis or flavonoid biosynthesis in organs other than petals.

Proteins likely to regulate flavonoid biosynthesis and epidermal cells in carnation were obtained by similarity searches against the assembled carnation sequences using amino acid sequences of reported MYB-TFs such as petunia An2, snapdragon MIXTA, petunia Odorant and Arabidopsis AtMYBL2 as queries.

The sequence data were retrieved from the DDBJ/EMBL/GenBank databases under the following accession numbers: GtMYB3 (AB289445), GtMYB1R1 (AB779612), GtMYB1R9, (AB779613), GtMYBP4 (AB289446), GtMYBP3 (AB733016) in *Gentiana triflora*; PhpH4 ((AY973324), PhAn2 (AF146702), ODO (AY705977), PhEOBII (EU360893) in *Petunia hybrida*; VvMYB5b (AB899404), VvMYBA1 (XM_003631456); VvMYBPA1 (AM259485), VvMYBF1 (FJ948477) in *Vitis vinifera* ; AtMYB16 (NM_121535), AtTT2 (NP_198405), At MYB75PAP1 (NP_176057), At AtMYB90PAP2 (NP_176813), ANT1 (AY348870); AtMYB111 (NM_124310), AtMYBL2 (NM105772), CPC (NM 130205); AtMYB11 (NM_116126), AtMYB12(AF062864); AtMYB106 (NM_110979), in *A. thaliana*; AmMIXTA (X79108), AmROSEA1 (DQ275529) and AmVENOSA (DQ275531) in *Antirrhinum majus*,; ZmP1(L19495) and ZmC1 (M37153) in *Zea mays*; LjTT2a (AB300033) in *Lotus japonicus*; GhMYB10 (AJ554700) in *Gerbera hybrida*; LhMYB6 (AB534587) and LhMYB12 (AB534586) in *Lilium* hybrid cultivar; OgMYB1 (EF570115) in *Oncidium* hybrid cultivar; PhMYB9 (FJ039863) in *Phalaenopsis* hybrid cultivar; InMYB2 (AB234211) in *Ipomoea nil*; MdMYB10 (EU518249) and MdMYB110a (JN711473) in *Malus x domestica*; FaMYB1 (AF401220) in *Fragaria x ananassa*.

a. Hichri, I., Barrieu, F., Bogs, J., Kappel, C., Delrot, S. and Lauvergeat, V. 2011, Recent advances in the transcriptional regulation of the flavonoid biosynthetic pathway, *J. Exp. Bot.*, **62**, 2465-2483.
b. Yamagishi, M., Shimoyamada, Y., Nakatsuka, T. and Masuda, K. 2010, Two *R2R3-MYB* genes, homologs of petunia *AN2*, regulate anthocyanin biosyntheses in flower tepals, tepal spots and leaves of Asiatic hybrid lily, *Plant Cell Physiol.,* **51,** 463-474.

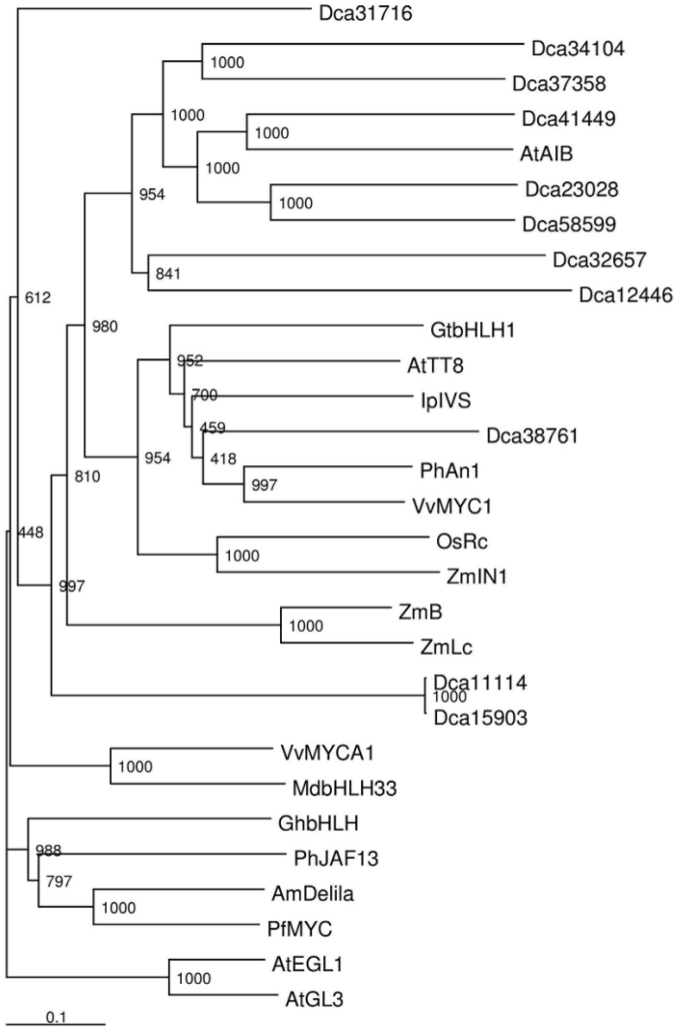

Supplementary Figure S12. Phylogenetic analysis of bHLH-type transcription factors related to flavonoid biosynthesis.

A total of 139 genes homologous to genes for bHLH transcription factors were assigned to the carnation genomic sequences, which is comparable to other plants; for example, there are 162 such genes in *A. thaliana*, 167 in rice and at least 119 in grapevine.[a] Eleven genes most similar to petunia *An1,* which is required for anthocyanin synthesis, and genes for bHLH-type TFs from other plants that regulate flavonoid biosynthesis, were subjected to phylogenetic analysis. Dca38761 is closely related to petunia An1 and *Ipomoea purpurea* ivory seed.

Genes that are likely to regulate flavonoid biosynthesis were similarity-searched using the reported amino acid sequence of petunia An1 as a query. Eleven genes with the highest similarity and genes for bHLH-type TF regulating flavonoid biosynthesis from other plants were included to construct a phylogenetic tree.

The genes analyzed were as follow: *A. thaliana* (AtTT8, NM_117050; AtEGL1, NM_001198373; AtGL3, NM_148067, AtAIB, NM_130216), *Antirrhinum majus* (AmDelila, M84913), *Oryza sativa* (OsRc, AB247503), *Petunia hybrida* (PhAn1, AF260919; PhJAF13, AF020545), *Vitis vinifera* (VvMYC1, EU447172; VvMYCA1, EF193002), *Zea mays* (ZmB, X57276; ZmLc, M26227; ZmIN1, U57899), *Malus x domestica* (MdbHLH33, DQ266451), *Gerbera hybrida* (GhbHLH, AJ007709), *Perilla frutescens* (PfMYC, AB024050), *Ipomoea purpurea* (IpIVS, AB154369) and *Gentiana triflora* (GtbHLH1, AB450661).

a. Hichri, I., Barrieu, F., Bogs, J., Kappel, C., Delrot, S. and Lauvergeat, V. 2011, Recent advances in the transcriptional regulation of the flavonoid biosynthetic pathway, *J. Exp. Bot.*, **62**, 2465-2483.

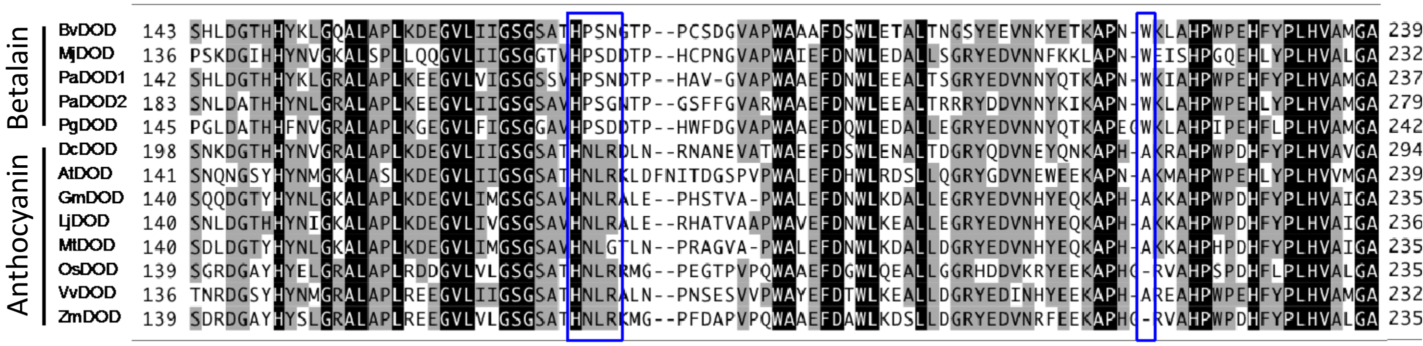

Supplementary Figure S13. Multiple alignment of conserved motif specific to DOD proteins.

Multiple alignment was carried out using the CLUSTALW algorithm. The amino acid sequences analyzed were as follows: BvDOD (*Beta vulgaris*, CAE47100), MjDOD (*Milabilis jalapa*, AB435372), PaDOD1(*Phytolacca americana*, AB451869), PaDOD2 (*P. americana*, AB451870), PgDOD (*Portulaca grandiflora*, Q7XA48), DcDOD (*Dianthus caryophyllus*), AtDOD (*A. thaliana*, NP_567456), GmDOD (Glycine *max*, XP_003548265), LjDOD (*Lotus japonicus*, AFK38970), MtDOD (*Medicago truncatula*, XP_003619705), OsDOD (*Oryza sativa*, NP_001044982), VvDOD (*Vitis vinifera*, XP_002283881) and ZmDOD (*Zea mays*, EU953437) .

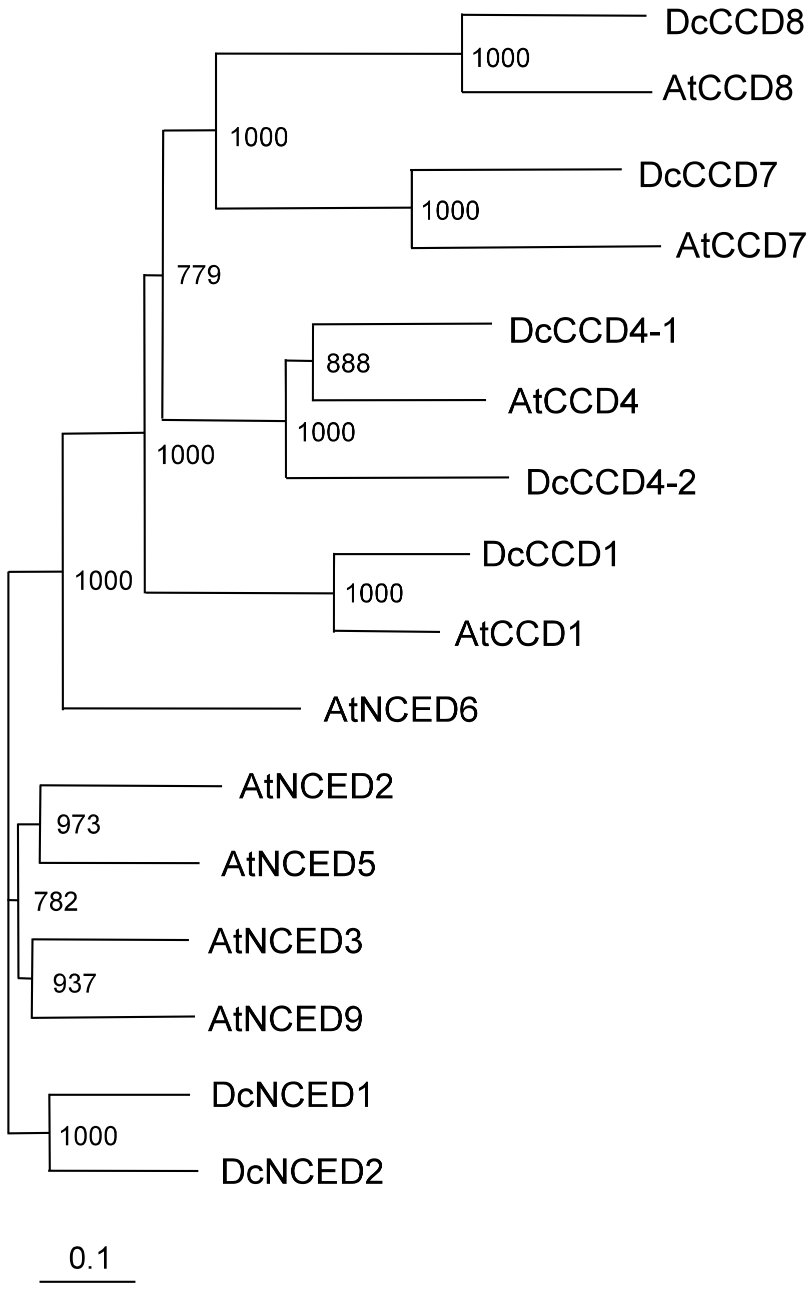

Supplementary Figure S14. Phylogenetic analysis of NCED and CCD proteins from carnation and *A. thaliana*.

Steady-state levels of carotenoids in plant tissues are, to a great extent, determined by the relative rates of biosynthesis and degradation.[a] *A. thaliana* has nine genes encoding carotenoid cleavage dioxygenases.[b] Five of these (NCED2, NCED3, NCED5, NCED6 and NCED9) are involved in abscisic acid biosynthesis, while the remaining four (CCD1, CCD4, CCD7 and CCD8) have different enzyme activities and substrate specificities. In carnation, two homologs of NCED and two homologs of CCD4, as well as single homologs each of CCD1, CCD7 and CCD8, were assigned.

Phylogenetic relationships were deduced using the neighbor-joining method. Numbers at branch points indicate bootstrap values (1,000 replicates). Scale bar, 0.1 amino acid substitutions per site.

a. Ohmiya, A. 2009, Carotenoid cleavage dioxygenases and their apocarotenoid products in plants, *Plant Biotechnol.*, **26**, 351–358.
b. Tan, B.C., Joseph, L.M., Deng, W.T., et al. 2003, Molecular characterization of the *Arabidopsis* 9-*cis*-epoxycarotenoid dioxygenase gene family, *Plant J.*, **35**, 44–56.

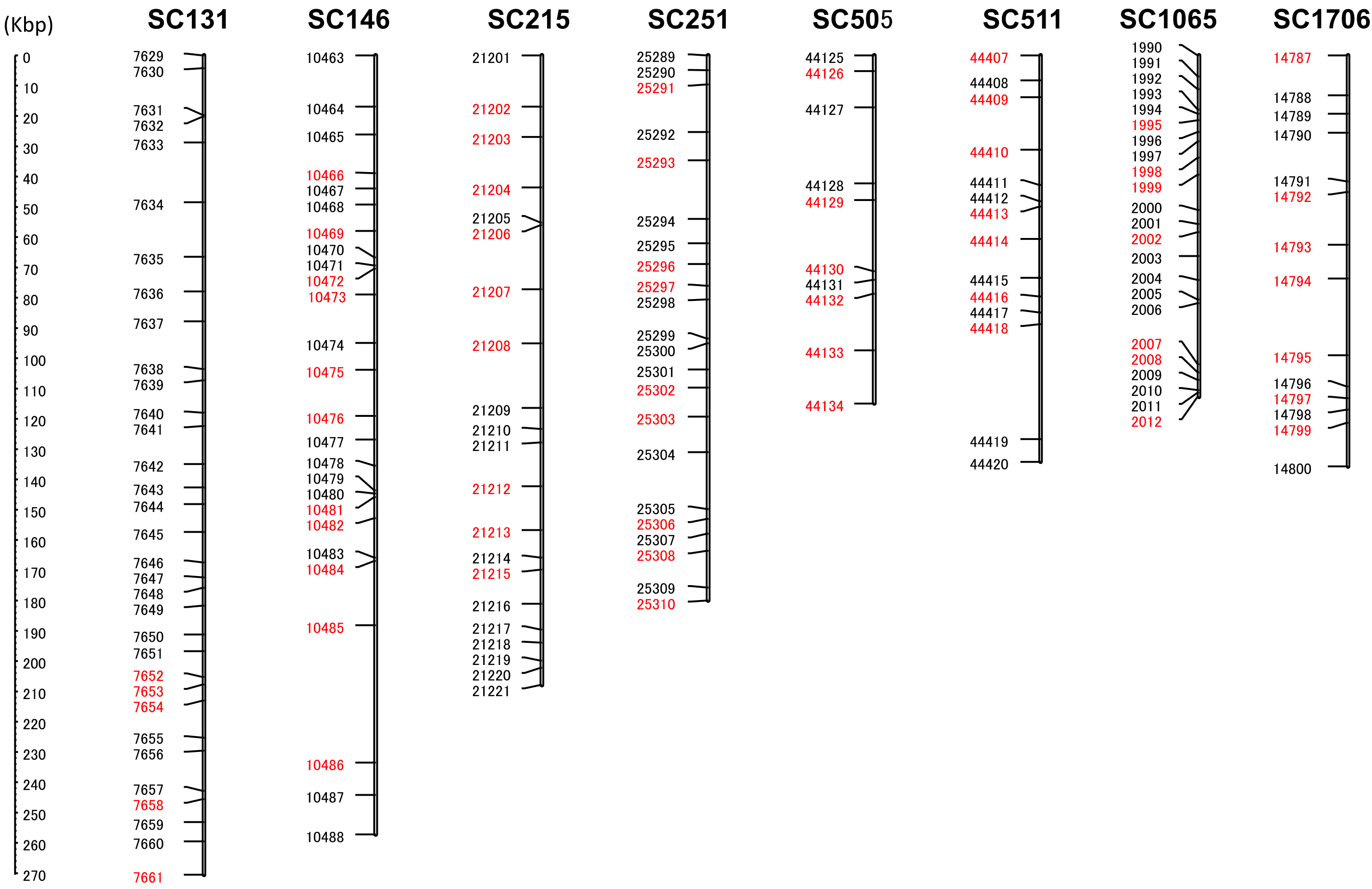

Supplementary Figure S15. Scaffolds containing five or more genes for NBS-containing proteins.

Vertical bar on the left shows the number of base pairs. Numbers in each scaffold (sc) indicate annotated genes in the carnation genome. Genes containing the NBS motifs are indicated in red.

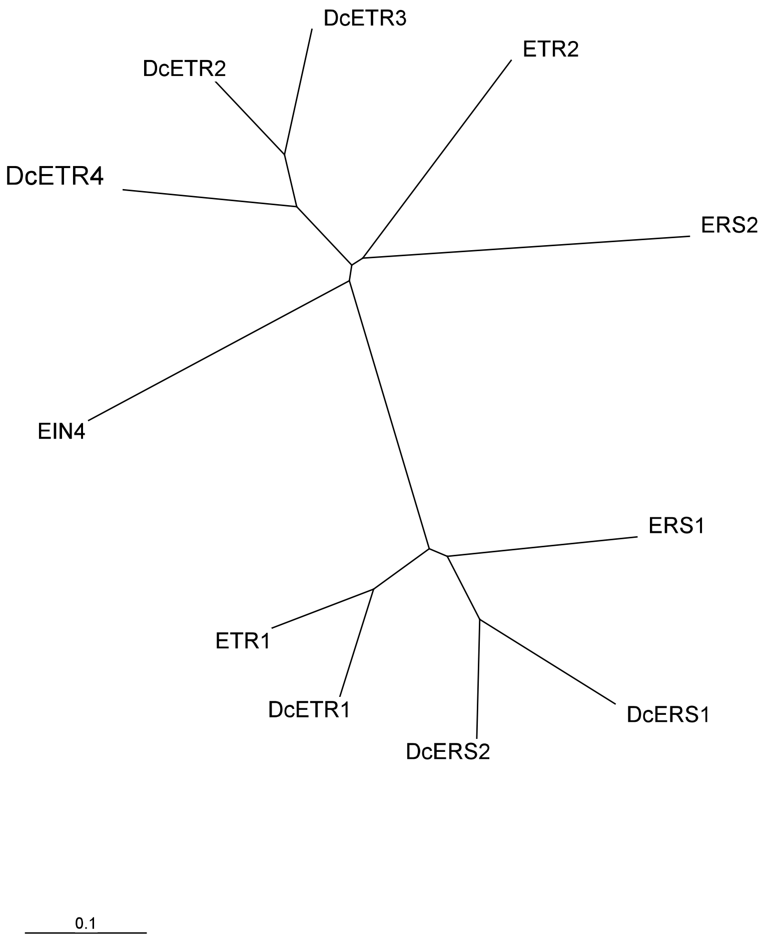

Supplementary Figure S16. Phylogenetic analysis of ethylene receptor genes in the carnation genome.

The evolutionary history was inferred using the Maximum Likelihood method based on the Tamura-Nei model.[a] Evolutionary analyses were conducted in MEGA5 and distance was calculated with default parameters.[b]

a. Tamura, K. and Nei, M. 1993, Estimation of the number of nucleotide substitutions in the control region of mitochondrial DNA in humans and chimpanzees, *Mol. Biol. Evol.*, **10**, 512-526.

b. Tamura, K., Peterson, D., Peterson, N., Stecher, G., Nei, M. and Kumar, S. 2011, MEGA5: Molecular evolutionary genetics analysis using maximum likelihood, evolutionary distance, and maximum parsimony methods, *Mol. Biol. Evol.,* **28**, 2731-2739.

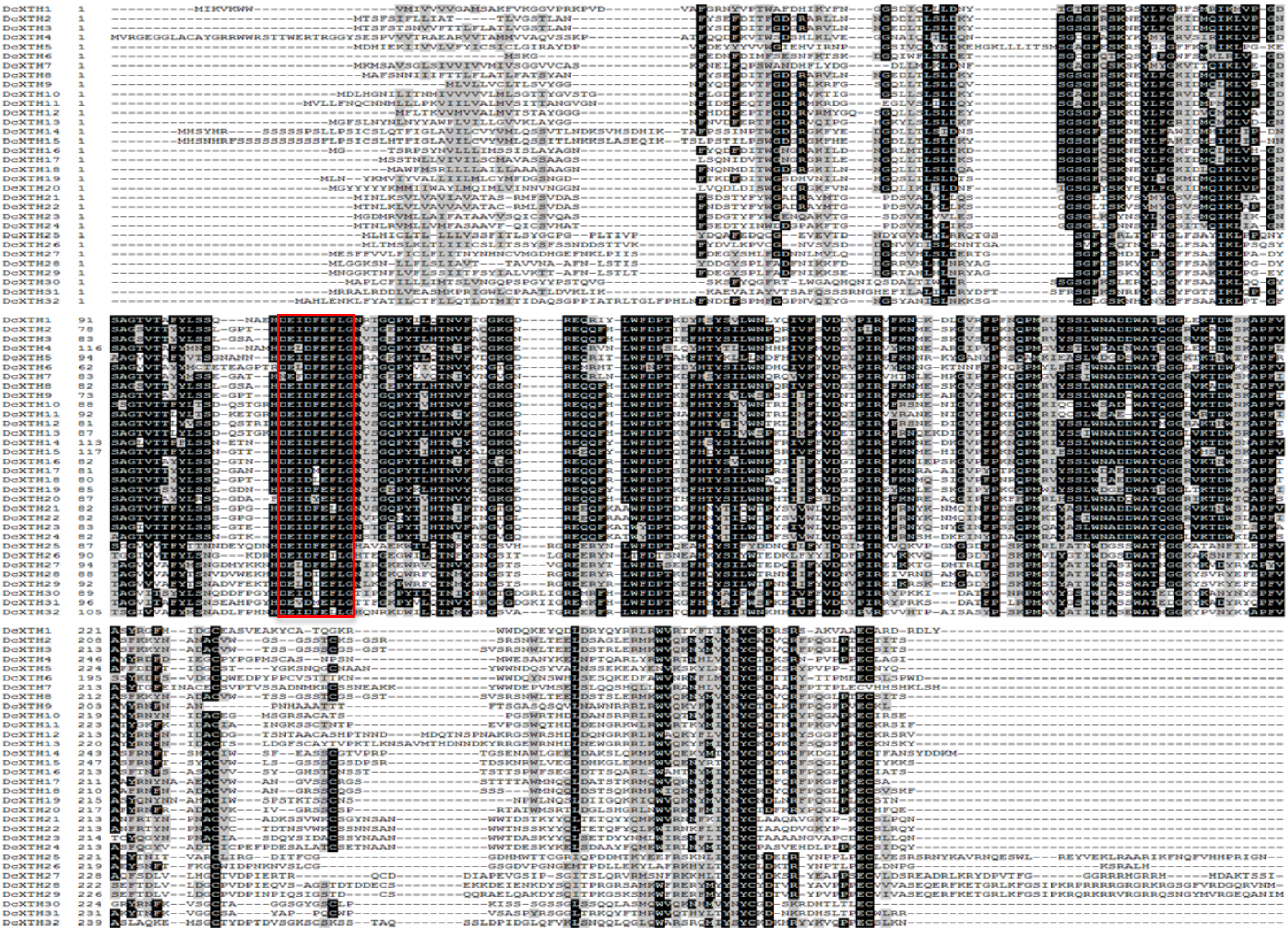

Supplementary Figure S17. ClustalW alignment of deduced amino acid sequences of xyloglucan endotransglucosylase/hydrolase (XTH).

Identical and similar amino acid residues are shaded in black and gray, respectively, and dashes indicate gaps. The DEIDFEFLG motif (highly conserved in XTH) is indicated by red box.

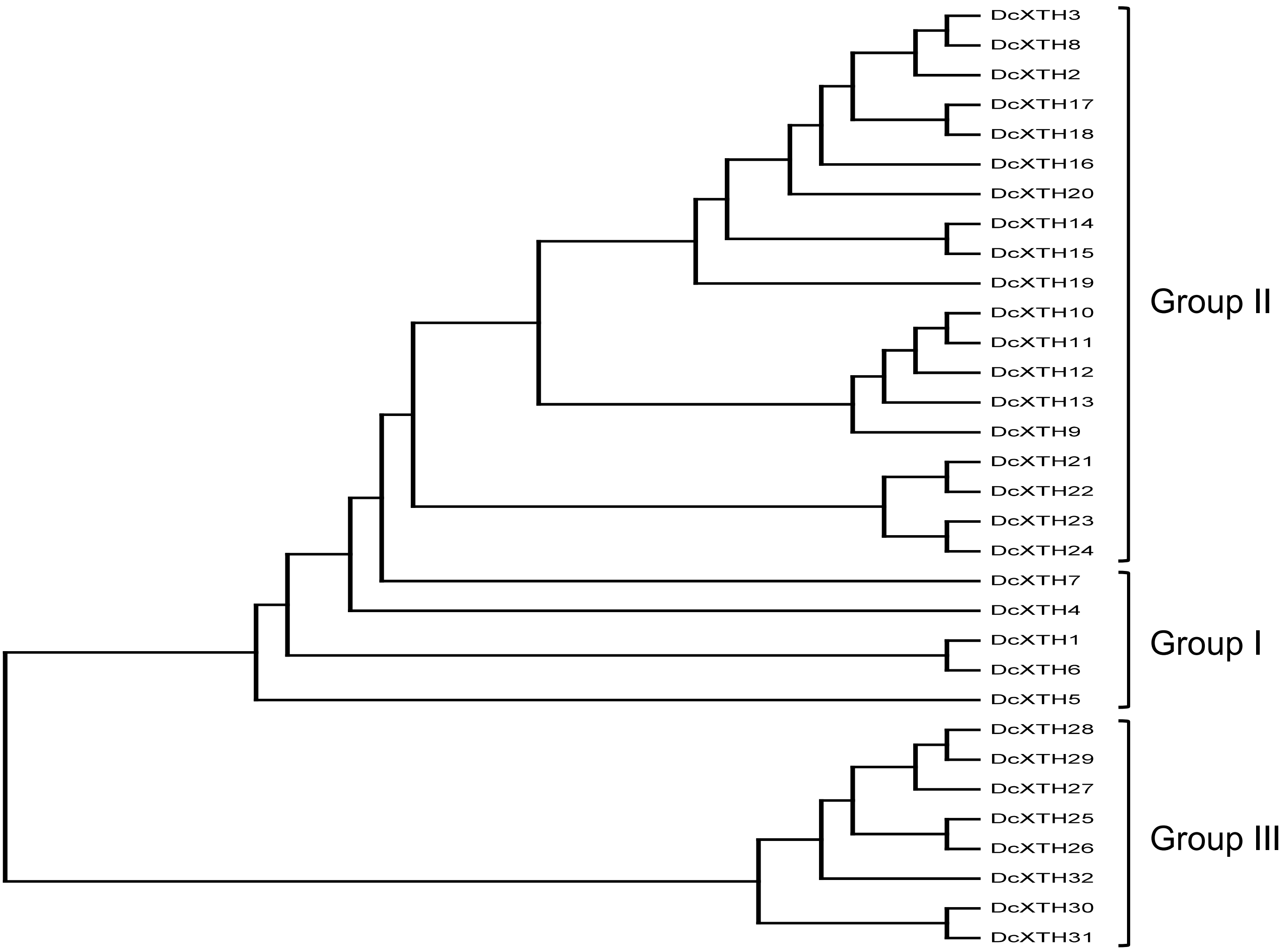

Supplementary Figure S18. Phylogenetic tree of xyloglucan endotransglucosylase/hydrolase (XTH). Amino acid sequences were aligned with CLUSTALW, and a phylogenetic tree was generated with TreeView program. All members were classified into three groups according to classification of *Arabidopsis* XTHs.[a]

a. Yokoyama, R. and Nishitani, K. 2001, A comprehensive expression analysis of all members of a gene family encoding cell-wall enzymes allowed us to predict *cis*-regulatory regions involved in cell-wall construction in specific organs of Arabidopsis, *Plant Cell Physiol.*, **42**, 1025-1033.

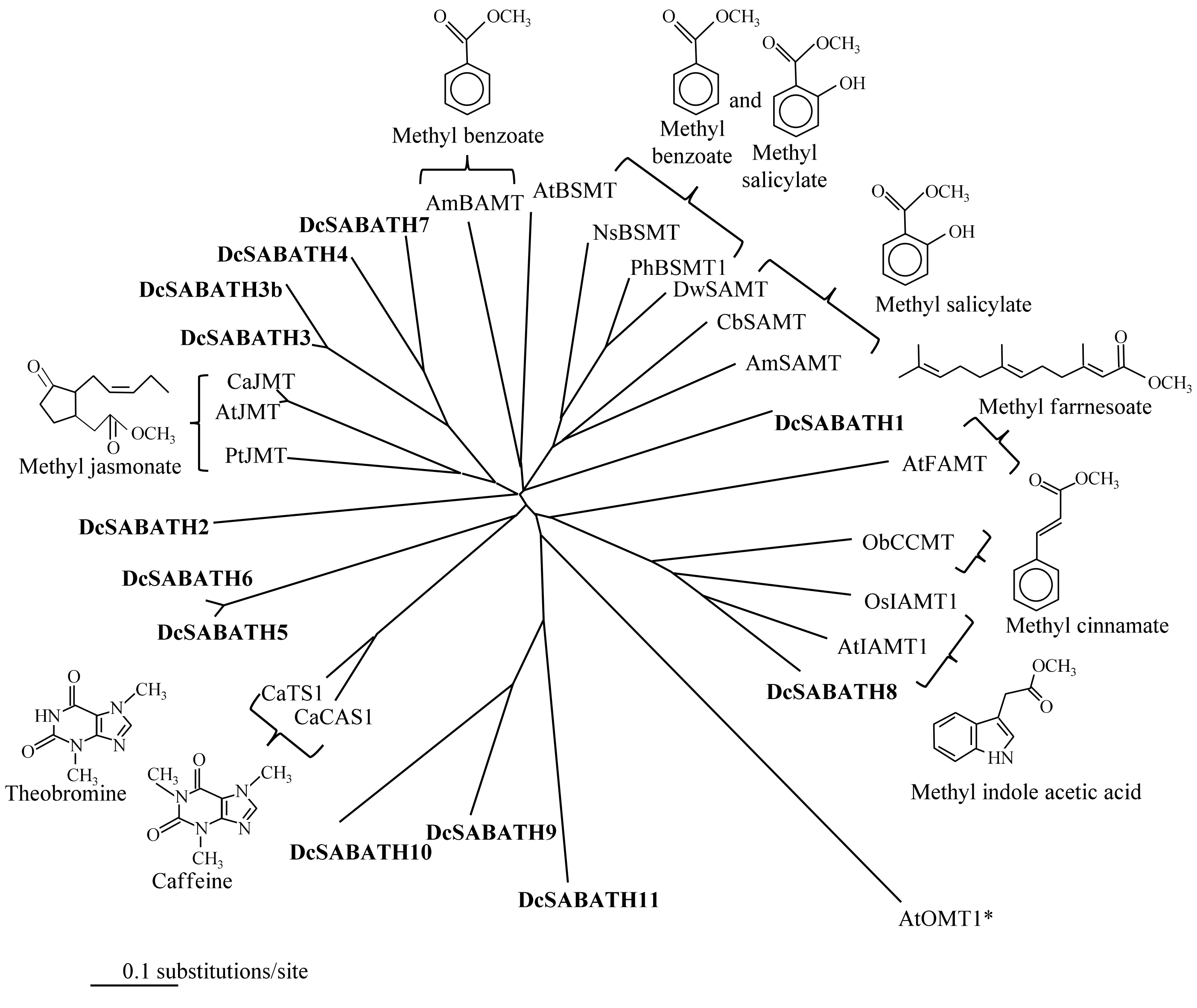

Supplementary Figure S19. Phylogenetic tree of carnation SABATHs (DcSABATHs) with SABATH family members in higher plants.

AmBAMT: *Antirrhinum majus* benzoic acid methyltransferase (AAF98284); AtBSMT, *Arabidopsis thaliana* benzoic acid/salicylic acid methyltransferase (AAY25461); NsBSMT, *Nicotiana suaveolens* BSMT (CAF31508); PhBSMT1, *Petunia hybrida* BSMT1 (AAO45012); CbSAMT, *Clarkia breweri* salicylic acid methyltransferase (AAF00108); AmSAMT, *A. majus* SAMT (AAN40745); DwSAMT, *Datura wrightii* SAMT(ABO71015); AtJMT, *A. thaliana* jasmonic acid methyltransferase (AAG23343); CaJMT, *Capsicum annuum* JMT (ABB02661); PtJMT, *Populus trichocarpa* JMT (AGR50489); AtIAMT1, *A. thaliana* indole acetic acid methyltransferase 1 (BAD43349); OsIAMT1, *Oryza sativa* IAMT1 (ABZ04474); ObCCMT1, *Ocimum basilicum* cinnamate/*p*-coumarate methyltransferase (ABV91100); CaCAS1, *Coffea arabica* caffeine synthase 1 (BAC43760);CsTCS1, *Camellia sinensis* theobromine and caffeine synthase 1(BAB12278).

* *A. thaliana O*-methyl transferase1 (AtOMT1) is not a SABATH family member.
